# Individual and generational value change in an adult population, a 12-year longitudinal panel study

**DOI:** 10.1038/s41598-022-22862-1

**Published:** 2022-10-25

**Authors:** Ingmar Leijen, Hester van Herk, Anat Bardi

**Affiliations:** 1 School of Business and Economics, Vrije Universiteit Amsterdam, Amsterdam, The Netherlands; 2 Royal Holloway University of London, Egham, UK

**Keywords:** Psychology, Human behaviour

## Abstract

A long-standing conundrum is whether age differences in personality are due to generation, or internal change with age. Using a representative sample from The Netherlands (N = 1599; aged 16–84 at the start), the current research focuses on human values (an important aspect of personality), following the same individuals for 12 years. We distinguish four generations, Silent-generation, Baby-boomers, Generation-X and Millennials. We found clear differences across generations in human values, with Millennials, e.g., valuing hedonism more than all other generations. Furthermore, value change over time was mainly evident in Millennials. Some values (achievement and conformity) were stable within individuals and between generations. Change over time across most values occurred mainly in Millennials, but not for all values. Some values were stable in adults (e.g., hedonism, conformity) while other values still increased (e.g., security, self-direction) or decreased (e.g., power, stimulation) in importance. In adults older than Millennials change decreased and change was absent in the oldest generation. Hence, age differences in values seem both due to generation, as well as internal change, although the latter mainly in young adults. These value changes over time may have implications for developments in societal values in the long run.

## Introduction

A general opinion on values and aging is that young people want to have fun and old people are conservative, implying that people’s values change with age. In this view, age differences are due to internal changes with age rather than generation differences. However, theory predicts that individuals’ values are rather stable over time, which contradicts this possible change in values^1^, raising the question if individuals’ values change as they age, or remain stable after a certain age, and it is birth cohort that is behind age differences in values? We examine this question using representative data from the Netherlands, and spanning four generations (Millennials, Generation-X, Baby-boomers, and the Silent-generation)^2^ over a 12-year period (2008–2020).

### Background

Values are abstract ideals that function as guiding principles in life^3^ and are considered a stable part of someone’s psychological profile, influencing attitudes, needs, and behaviors^1^. Values are considered relatively stable over time^4^. Unlike attitudes or needs, they do not easily change with circumstances. Examples of values that are important to most people are, for instance, caring for other people and having freedom^5^. Cross-sectional studies on human values show that value priorities of younger and older individuals differ^6–8^. These studies involved distinct samples such as students and teachers^9^, representative samples^10^, and large cross-national studies^11,12^. Value differences between generations (e.g., Baby-boomers, Generation-X, Millennials) have also been shown in several studies^2,13^. These studies consistently showed that values differ between age groups, with younger age groups considering openness values more important and older age groups conservation values. Recent work shows value differences across the life span reflect a universal pattern across different countries^14^. However, it is possible that the results that were found were due to generational differences. To obtain insight into changes over time in specific age groups, differences between individuals at different points in time may be approximated using a meta-analysis^15^. However, to confidently determine whether age or generation (i.e., birth cohort) drives value differences across ages, a longitudinal study is required including individuals at different points in time, over a long period.

Recent work recognizes this, resulting in intra-individual value change being an emerging topic in psychological research^16^. These studies can be categorized to three types: First, experiments changing values in the short-term in the laboratory^17,18^. Second, studies including two points in time and including significant life events. In these studies, the focus is on intra-individual value change due to events like an earthquake^19^, a terrorist incident^20^, immigration^21,22^ as well as life stage specific events such as attending university^23^, or becoming a parent^24^. Third, longitudinal studies at more than two time points. E.g., Vecchione, et al.^25^ who studied 107 young adults (21–22 years old at start) over a period of 8 years, and Milfont, et al.^26^ who used a large representative sample of 3,434 adults (aged 25–71 at start) over a period of 3 years. However, as values are considered rather stable aspects of people’s personality, even 3 years is still a relatively short period for measuring intra-individual value change. Moreover, according to theory, most value change happens during the younger life stages^27^, thus limiting the insights of this study.

### Schwartz human values

Currently the most widely used framework of human values is by Schwartz^3^. He conceptualizes values into 10 different value-types that together form a *value-circumplex*, with two main dimensions underlying the differences between values. The first dimension opposes the value domain Openness-to-change (self-direction, stimulation) with the value domain Conservation (conformity, tradition, security). The other value dimension is the opposition between Self-enhancement value-types (power, achievement) versus Self-transcendence value-types (universalism, benevolence). The value-type hedonism is situated in between the Self-enhancement and Openness-to-change poles. Value-types can either be more in accordance with each other (the closer they are on the circumplex, the stronger they correlate) or more in conflict with each other (being on opposing sides of the value-circumplex). This means that a higher priority of a certain value-type is expected to be associated with a lower priority of a value-type on the opposing side of the circumplex.

### Value change and stability

After value priorities have developed during childhood, they are considered relatively stable over the life span^3,4^. However, “relative” leaves room for change: research into value change suggests that value change after childhood is possible, and that these changes could be attributed to a host of factors. Schwartz^28^ mentions 3 potential sources of value change in adults: 1; societal events like war, famine, economic crisis, pandemics, 2; aging-related physical decline, and 3; life events like leaving school, marrying, becoming a parent, retirement.

There are large cross-sectional studies using representative samples that include the relation between age and value priorities. Schwartz^28^ concluded from a review that age is positively correlated with conservation and self-transcendence, and negatively with openness-to-change and self-enhancement. The capability of recognizing, and distinguishing between values, was already found in children as young as 5^29^. Research using European Social Survey data found older cohorts leaning more to conservation values and lower self-enhancement values, and younger cohorts showing the opposite^11,12,30^. However, if measured cross-sectionally, differences between generations cannot be attributed to either age or cohort: an older generation would have experienced other societal events than a younger generation, but an older generation would have also experienced many more individual events and increased physical decline. Thus, it is impossible to attribute changes to just one of these sources using cross sectional data or specific age groups.

Longitudinal studies showed that the stability of children's values increases during childhood: the older the child, the higher the value stability^31,32^. For children in middle childhood self-transcendence and openness-to-change values became more important, while conservation and self-enhancement decreased in importance over time^32,33^. Other longitudinal studies showed that in early adolescence values were moving towards more importance for the value-domains self-enhancement and openness-to-change^34^, and for young adults the value-types self-transcendence, conservation, and power increased in importance, and achievement values decreased^25^. Graduating students increased in conformity and security and decreased in hedonism and self-direction values^33^. Finally, adults (aged 25–71), followed over a 3-year period, were found to increase in self-transcendence and conservation and decrease in openness-to-change^26^.

To summarize, these findings suggest that ageing in adults positively relates to conservation and self-transcendence values and negatively to openness-to-change and self-enhancement values. However, with existing research the question whether this is a change over time within individuals, or whether value change is a generational shift, cannot be answered. Researchers either investigated individual value change over brief time periods or only within limited age groups (e.g., children, adolescents, young adults), or used cross-sectional designs. Hence, the question of whether individual value change is also possible over the entire lifespan has been theoretically addressed but lacks empirical evidence with a representative sample including the same people over a longer period of time.

Our longitudinal approach, in a large Dutch sample, provides insight into values people have at the start of the study as well as in the consecutive development of their values over a 12-year period, investigating change in people from late adolescence to the elderly. We distinguish 4 generations, the first born before WW2, the second growing up in the post-war reconstruction years, the third in an era of increasing economic growth, and the last experiencing a period of high prosperity^35,36^.

Corroborating existing cross-sectional research, we show the differences between younger and older people in value importance. For this we classify them into known generations (i.e., Silent-generation, Baby-boomers, Generation-X, Millennials) and show that there are meaningful differences. We address several aspects of value stability and change. First, we investigate value-profile stability: the stability (within individuals) of the relative order of importance of values. We compare the average value-profile stability between four different generations. Next, we analyze the stability of each value-type: we compare the rank-order stability of each value-type over a 12-year period, again for each generation. And finally, we analyze the development of the relative (i.e., mean-level) importance of each value-type over time, for each generation.

## Results

### Value profile stability

**Figure 1:**
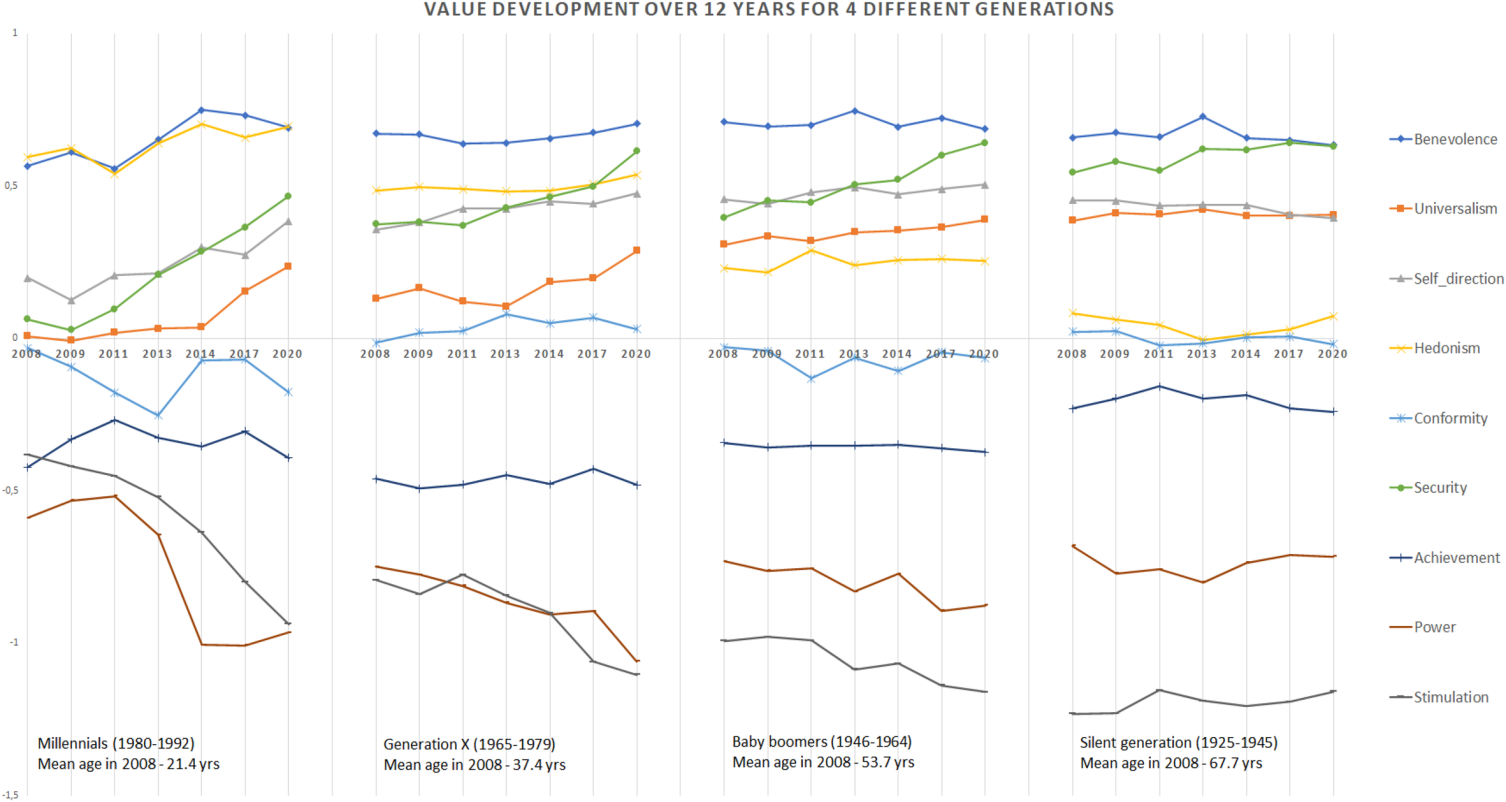
Mean value change for different generations over a period of 12 years. Lines shows change over time (7 measurements over a period of 12 years) in the relative importance of nine human value-types for four different generations (Millennials, Generation-X, Baby-boomers and Silent-generation. Scores are ipsatized means of the nine value-types, calculated per generation. Distances per time period (i.e., varying between 1 and 3 years) are equally spaced. Mean age of each generation is indicated for T0 (2008).

We start with the question: Does the order of importance people attach to the 9 values change over time? If value importance changes within individuals, one type of such change is change in the relative order of importance of the values that is, their personal value hierarchy. The more the personal hierarchies change, the lower the correlation between the value-profiles in different points in time. We assessed value-profile stability between 2008 and 2020. We calculated similarity of the individual value profiles using Spearman rank-order correlations; a value-profile stability of 0.00 indicates no relationship and a profile stability of 1.00 indicates the same rank-order after 12 years**.** Table 1 shows that Millennials were lowest and the Baby-boomers highest on value-profile stability, with Generation-X and the Silent-generation in between (*F*_(3,1576_ = 12.44, *p* = 0.000). A mean-comparison test between the generations using TukeyHSD shows that the average within-person value-profile stability is similar for Baby-boomers and the Silent-generation as well as for Millennials and Generation-X. The largest differences are between the Millennials and Silent-generation and between Millennials and Baby-boomers (See also SI 2 Fig. 1).

**Table 1:** Ipsative stability of the within-person value-type hierarchies (i.e., value-profile stability) in the period 2008–2020 within 4 generations and for all individuals.

Generation	Central tendency		Dispersion			Percentiles		Shape	
	Mean	Median	SD	Min	Max	25th perc	75th perc	Skewness	Kurtosis
Silent-generation	*.618*	.732	*.356*	−.911	.996	.515	.864	−1.862	3.748
Baby-boomers	*.643*	.722	*.283*	−.958	1.000	.519	.849	−1.613	3.476
Generation-X	*.562*	.655	*.330*	−.970	1.000	.402	.798	−1.476	2.665
Millennials	*.477*	.508	*.356*	−.895	.983	.292	.765	−.907	1.034
*Average across all generations*	*.610*	*.702*	*.316*	−*.970*	*1.000*	*.470*	*.839*	−*1.613*	*3.281*

Significant values are in italics.

Hence people’s values were quite stable over time, with most change found in the youngest generation, and least change in the Baby-boom generation. We also noticed a possible decline in value stability for the oldest generation.

### Value stability over time

A second aspect of value change is the stability of value priorities: how stable are people within a group regarding the priority of a certain value. If most people in a group have a similar score of the priority of a value across two time periods, correlations between these two time periods will be high, i.e., the stability of the value will be high. And vice versa: the more people within a group change in their appreciation of a particular value over time, the lower the correlation between two time periods and thus the lower the stability of this particular value in the population. To estimate how value priorities change over time for each value-type between all consecutive time points, we calculated rank-order correlations. To capture the ordinal character of the values measurement, we used Spearman correlations^37^. Results are shown in Table 2.

**Table 2:** Rank-order stability (Spearman rank-order correlations) in full sample of each human value over time between pairs of waves.

	2008–2009	2009–2011	2011–2013	2013–2014	2014–2017	2017–2020	2008–2020
Benevolence	.570	.572	.582	.590	.573	.572	.452
Universalism	.556	.582	.596	.595	.550	.572	.468
Self-direction	.562	.534	.566	.558	.531	.544	.480
Stimulation	.595	.576	.566	.588	.570	.564	.466
Hedonism	.499	.472	.491	.516	.510	.472	.432
Achievement	.471	.476	.477	.512	.481	.496	.408
Power	.449	.444	.493	.491	.464	.477	.377
Security	.577	.587	.596	.587	.566	.551	.454
Conformity	.507	.521	.504	.506	.483	.482	.432
Average across all values	.532	.529	.541	.549	.525	.525	.441

Columns indicate Spearman rank-order correlation coefficient for each value-type between 2 waves: Time between waves varies from 1 to 3 years. Last column indicates coefficient between first and last wave (12-year period). All correlations are significant *p* < .01.

Across all respondents the Spearman rank-order correlation between the respective value-types at two consecutive time points was on average *ρ* = 0.533, and *ρ* = 0.441 over the 12-years period (between 2008 (T0) and 2020 (T6)). Across all generations, between T0 and T6, self-direction was the most stable over time (*ρ* = 0.480) and power the least stable (*ρ* = 0.377).

To provide insight into differences between generations, we compared value stability across generations. Results are shown in Table 3.

**Table 3:** Rank-order stability (*Spearman rank-order correlations)* within generations of each human value over time between 2008 and 2020.

	Silent gen	Baby B	Gen. X	Millennials	Total sample
Benevolence	.424	.486	.464	.248	.452
Universalism	.454	.474	.458	.413	.468
Self-direction	.401	.573	.460	.270	.480
Stimulation	.396	.475	.505	.433	.466
Hedonism	.349	.388	.436	.299	.432
Achievement	.386	.445	.376	.250	.408
Power	.365	.389	.377	.354	.377
Security	.436	.468	.448	.372	.454
Conformity	.378	.483	.356	.423	.432
Average across all values	.399	.465	.431	.340	.441

Columns indicate Spearman rank-order correlation coefficient for each value-type between waves 1 (2008) and 7 (2020), per generation: Time between waves is 12 years. Last column indicates coefficient between first and last wave (12-year period). All correlations are significant *p* < .01.

As can be seen in this table, average value stability was clearly lower for the Millennial-generation and the highest average value stability was found within the Baby-boomer generation. Millennials were most stable in stimulation, conformity, and universalism values, and least stable in self-direction, achievement, and benevolence. Within the Baby-boom generation, the highest stability was found for self-direction.

To summarize, we found variation in stability of value-types. The highest stability in the total sample was found for the value-type self-direction, and lowest for power. Focusing on generations, overall lowest stability was found in the Millennial-generation, and the highest stability in the Baby-boomer generation. Within the youngest generation, values that were least stable were self-direction, achievement, and benevolence, while stimulation, conformity and universalism were most stable. Interesting to note is the instability of self-direction in the youngest generation, as self-direction is overall the most stable value.

### Mean value change over time

Although rank-order stability analysis showed that some values change over time and between generations, it does not give us insights into the direction, nor the scope in which values change. Figure 1 visualizes the development of the values over the period 2008–2020 for each generation respectively. This figure suggests more change over time within the Millennials compared to the other generations.

**Figure 2:**
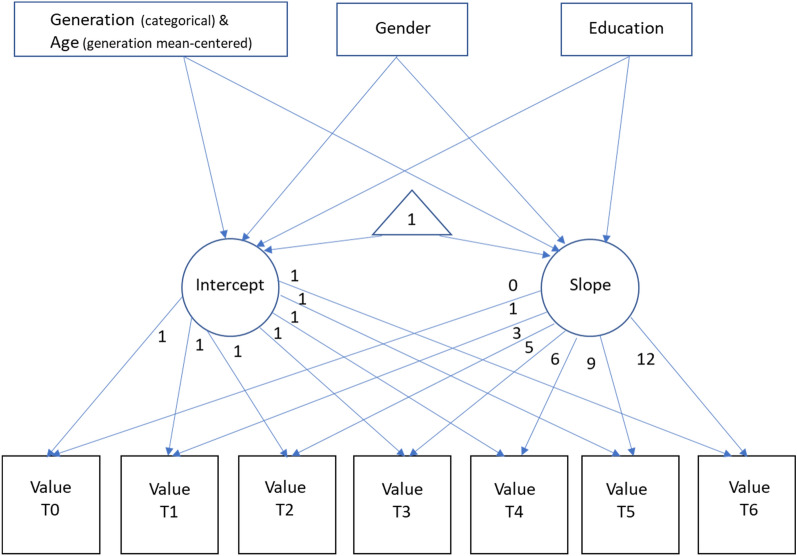
Latent growth model value change over 12 years with latent intercept and slopes.

To evaluate the validity of these changes we estimated latent growth curve models (LGCM)^38^, an established technique to assess within individual changes over time in longitudinal data^39^. To estimate the models, we employed the structural equation approach using lavaan in R^40,41^, including seven time periods (2008, 2009, 2011, 2013, 2014, 2017, and 2020) and three time-invariant covariates, gender (male = 1 female = 0), generation (dummy coding), education (high = 1, low = 0), and within generation age differences in 2008. These covariates were selected as previous literature has shown that age, gender and education are the most influential covariates in human values research^6^. For a visualization of the LGCM model and its parameters see Fig. 2.

For each value we estimated a latent growth curve model using maximum likelihood and 1000 bootstrap draws. In the analyses, we coded the times as 0, 1, 3, 5, 6, 9, and 12 indicating the difference in years between the time points. We tested several nested models (see web appendix for code), starting with intercept only, and intercept and slope only, followed by models adding the time-invariant covariates. As the variable measuring generations is categorical, we used the Silent-generation as the reference category.

All models converged without problems; common fit statistics including CFI, RMSEA and SRMR indicate that all models fit well using the expected criteria, i.e., > 0.95 for CFI, < 0.08 for RMSEA and SRMR^42^. By adding the time-invariant covariates, we assessed whether these covariates explained differences in intercept (mean-level differences in value priority) and differences in slopes (mean-level changes over time) of the individual value trajectories. For clarity, we only report the model with random intercept and slope and including all time-invariant covariates for each value. For all values the models with random slope and random intercept, including covariates fitted well (see supplemental material for fit statistics, SI 1 Table 1), with the worst fitting model (universalism) still fitting the data well with chi-square (59) = 188.10 *p* = 0.000, CFI = 0.979; RMSEA = 0.037; and SRMR = 0.029.

The results for all values are shown in Table 4 (see SI 11 Tables 6.1 to 6.3) for p-values and confidence intervals). Each column first shows the intercept across all respondents, indicating the overall relative importance of the value across all respondents, followed by the coefficients for the time-invariant covariates. For example, a positive coefficient for gender indicates that males score above average (i.e., the intercept) on the value. Next, the overall slope (indicating change over time) is reported, again followed by the coefficients for the covariates. As the Silent-generation is treated as the reference category in the model, coefficients for this generation are not shown (i.e., being 0.0). The coefficients for the other cohort are to be interpreted in comparison to the reference category. We now report the results for each of the values, starting with benevolence.

**Table 4:** Growth curves per value; no change and linear change; SEM approach lavaan (R; Rosseel, 2012).

	Benevolence	Universalism	Self-direction	Stimulation	Hedonism	Achievement	Power	Security	Conformity
Intercept mean	0.767***	0.485***	0.434***	−1.392***	0.050	−0.327***	−0.924***	0.744***	0.163***
Gender (male = 1)	−0.185***	−0.200***	−0.062*	0.393***	0.014	0.091***	0.263***	−0.188***	−0.125***
Education (high = 1)	0.050*	0.091***	0.174***	−0.144**	−0.028	0.261***	0.093*	−0.247***	−0.250***
Age°	0.002	0.011***	0.002	−0.023***	−0.015***	0.002	−0.001	0.012***	0.009*
Baby-boomers^1^	0.014	−0.108***	−0.014	0.279***	0.192***	−0.161***	0.025	−0.155***	−0.073
Generation-X	−0.054	−0.323***	−0.107**	0.517***	0.435***	−0.307***	0.014	−0.199***	0.023
Millennials	−0.138**	−0.498***	−0.393***	0.977***	0.554***	−0.271***	0.201	−0.433***	0.001
Slope mean	−0.005	−0.001	−0.005*	0.008	0.001	−0.003	0.007	0.003	−0.005
Gender (male = 1)	0.004*	0.002	0.000	−0.007	−0.006**	0.003	−0.007	0.005*	0.006*
Education (high = 1)	−0.001	0.002	0.001	0.001	0.003	−0.005	−0.007	0.006**	−0.002
Age°	0.000	0.000*	−0.001**	0.001**	0.000	0.000	0.000	0.000*	0.000
Baby-boomers	0.003	0.006*	0.009***	−0.022***	0.003	0.002	−0.015**	0.012***	0.002
Generation-X	0.006	0.011***	0.014***	−0.033***	0.004	0.005	−0.024***	0.011**	0.007
Millennials	0.017*	0.018***	0.022***	−0.054***	0.009	0.005	−0.043***	0.027***	−0.001
Intercept variance	.157	.123	.173	.609	.145	.170	.443	.211	.283
Slope variance	.0003	.0003	.0004	.0015	.0003	.0005	.0017	.0006	.0005
Covariance intercept-slope	−.0016	−.0008	−.002	−.009	−.0005	−.003	−.003	−.004	−.003

Associated *p*-values and confidence intervals can be found in the supplementary materials (SI 11, Table 6.1—6.3).

*p < .05; **p < .01; ***p < .001.

^1^Silent-generation is the reference category. ° mean-centered within generation (i.e., age means age difference within generations).

*Benevolence*, the most important value overall, was a less important value for men than for women, and slightly more important for the higher educated, no effects were found for within generation age differences. We only found significant differences for the Millennials: benevolence had a significantly lower priority compared to the Silent-generation and increased slightly in the 12-year period. Men showed a minor increase over time in benevolence.

*Universalism* was less important for men and more important to the higher educated, and older people were higher on universalism (within each cohort). As for the differences between the generations, Baby-boomers, Generation-X and Millennials, scored lower on universalism compared to the Silent generation, with Millennials scoring lowest. If we look at value change, we see that generations increased in universalism over time with the largest increase in the Millennial-generation.

*Self-direction* was more important for females and higher educated. Relative to the Silent-generation, self-direction was significantly less important for Generation-X and Millennials. Regarding change in value importance over time, the importance of self-direction increased over the 12-year period for the Baby-boomers, Generation-X and most for Millennials.

*Stimulation* was more important to males and less to the higher educated. In each generation, older people scored lower on stimulation. Relative to the Silent-generation, stimulation was more important to all other generations, with the Millennials having a significantly higher score than all other generations. Regarding value change over time, for Baby-boomers, Generation-X, and Millennials stimulation decreased in importance, with Millennials decreasing most.

For hedonism we found no significant differences for gender nor education and older people within each generation scored lower on hedonism. The importance of hedonism was higher in all generations compared to the Silent-generation, with the Millennials considering hedonism more important than the other generations. Over time none of the generations changed in their appreciation of hedonism.

*Achievement* was more important for men, more important to the higher educated, and being older was related to a higher achievement score. Significant mean differences in achievement were visible: Compared to the Silent-generation, all generations were lower on achievement. We found no significant change over the 12-year period for any of the generations, which contradicts previous cross-sectional findings^11,28^.

In line with literature power was much higher for men than women. Also, a higher educational level was positively related to this value. Relative to the Silent generation, Millennials valued power more. Notwithstanding the lack of difference of the other generations with the silent generation, we found Millennials decreasing most over time.

The value of security was the second most important value overall. Men attached less importance to security, and higher educated also valued security less. Within each generation older people scored higher on security. In all generations (compared to the oldest generation), security was lower, with Millennials scoring lowest. The value‐type security seems less stable over time compared to most other value‐types: Men, over the 12‐year period, increased slightly in security, as did the more highly educated. The different generations did all increase significantly in security, with the Millennials changing most, but also Generation X and the Baby‐boomers showing an increase over time.

Conformity was lower for men, and for the more educated. There were no significant differences between the generations, nor did the value change over time for any of the generations.

To summarize the results of the latent growth curve models, we found differences in intercepts between generations for all values except for conformity. Differences for benevolence and power were only minor: only the youngest generation scored significantly lower on benevolence and higher on power. Universalism, self-direction and security were less important in younger generations, while hedonism and stimulation were more important in these generations.

The coefficients indicating change (slopes) show that, within generations, values did not change to the same extent or in the same direction. The main change was in the Millennials, with benevolence, universalism, self-direction, and security increasing, while stimulation and power were decreasing. For Generation-X and Baby-boom generation, the largest changes were in stimulation and power. Over time, hedonism, achievement, and conformity did not change in any of the generations. The latter value-types were stable over a 12-year period. Overall, the largest changes over time were seen in the Millennial-generation (see also Fig. 1).

## Discussion

Our study adds important insights into the long-standing conundrum of whether age differences in values are due to generation differences or internal changes with age. We examined the way human values changed over a 12-year period in a representative Dutch sample of 1,599 people. We examined value change over time within individuals and explained differences between individuals by the generation they are in. Our approach differed from previous research into value change: previous research on value change mainly employed cross-sectional studies, and longitudinal studies on value change (until now) were confined to shorter time spans and/or non-representative samples^26,43^. These studies showed an increase in the value domains conservation and self-transcendence, and a decrease in the value domains of openness-to-change and self enhancement with older age.

Across four generations covering people aged 16–84 at the start, we found that older generations (Silent-generation and Baby-boomers) gave higher importance to universalism, achievement, self-direction, and security, and lower importance to hedonism and stimulation (compared to Generation-X and Millennials). We found that values changed most in the youngest (Millennials) generation. In this generation we found lowest stability, both in individual value-profile, but also in the rank-order of each value-type. Value-profile stability was highest within the Baby-boom generation. Interestingly, we also noticed a (statistically non-significant) decline in stability within the Silent-generation (see Table 1), suggesting a possible curvilinear relation of age and value stability. It could be that the confrontation with one’s mortality, which is often less salient for the young is influencing people’s value priorities^44^. This would be in line with a study in personality research showing that within an elderly sample (aged between 69–81), the oldest group showed personality change while the younger group did not^44^. However, it is also possible that the lower stability is due to age related cognitive decline, leading to some people in the generation becoming less reliable in understanding the survey questions^45^.

Regarding value change over time, we found that rank-order stability in the total sample differed per value-type: in the full sample, power was lowest, and stimulation was highest in stability, corroborating other research findings^23^. Our dataset spanning 12 years enabled us to show that value stability was not similar across generations, with the youngest (Millennial) generation being least stable. Specifically, within the Millennial-generation self-direction, achievement, and benevolence were less stable compared to the other generations. The lower rank-order stability of these three value-types within the Millennials was consistent with the lower value-profile stability of this group and may point to a further developing value awareness in younger people, as has also been shown with adolescents^46^. A development in value consciousness is also in line with the increasing perceptual distance between the values for the youngest generation (see Fig. 1); for instance, over the 12-year period, security and self-direction became more, and stimulation and power became less important, consistent with a maturation^47^ perspective. For the youngest generation becoming a parent could be a trigger for changes in their value-profile as the presence of children can invoke the promotion of prosocial values^48^.

In our results concerning mean value change one value stood out; we found the oldest generation caring least, and the youngest generation caring most for hedonism, however, the value of hedonism did not change over the 12-year period in any of the generations. Thus, we saw a change between generations, but not over time within the generations (see also Supplementary material SI 12 Table 10, 11). This result contrasts with results from Schwartz^28^, who proposed that hedonism declines with age. In view of our results, his results could instead be attributed to generational changes instead of age.

Looking at the pancultural hierarchy of values^5^ hedonism belongs to the least important values, however for our Dutch sample we found hedonism to be one of the more important values. For the Millennial-generation in our sample hedonism is even among the 3 most important values, while for the Silent-generation it’s among the least important values (Fig. 1).

Within Dutch society the increase in hedonism between generations may be linked to increasing wealth and attention to child rearing: the number of children has decreased over time^49^, while the amount of attention that is given to children has increased. Dutch culture attaching importance to a fun and relaxed childhood could be a cause why hedonism is increasing over generations and may be a reason that Dutch children are amongst the happiest in the world^50^.

In younger generations we found changes over time, but with each subsequently older generation these changes become smaller. Thus, in line with previous research, human values seem to become engrained with age. However, we also found that not all values become stable in a similar manner. Although values changed most in the Millennial-generation, already some values were relatively stable (hedonism, conformity, achievement), while other values (benevolence, universalism, self-direction, stimulation, power, and security) changed over a 12-year period within the youngest group. Despite changes over time becoming smaller in older generations, we found some values may be still somewhat malleable during adulthood (see also SI 11, table 6.1–6.3).

Within our Dutch sample, we found an increase in security and universalism and a decline in the need for stimulation in all generations over time. As the Netherlands is an affluent, democratic, high trust society^51^, changes and differences in values should be interpreted in this context. For instance, the (comparatively) low priority of power and high priority of self-direction are reflective of Dutch society^52^. Societal events and trends could have caused period effects: e.g., the aftermath of the credit crisis of 2008, the influx of workers from Eastern Europe, increased fear for terrorism (sparked by for instance the attack on the royal family in 2009, and a mass shooting in a mall in 2011), an ongoing refugee crisis, but also (and maybe related) increasing populism, increasing income inequalities, the climate crisis and strong societal discussions on universalist versus nationalist sentiments.

In summary, within our current research mainly the youngest generation showed indeed change over time for most values, although not for all. Some values seem more stable in adulthood (achievement, conformity, and hedonism) while other values still may increase (security, self-direction, universalism, and benevolence) or may decrease (power, stimulation) in importance. In adults older than the Millennial-generation there was value change (in particular for security, stimulation, and universalism), however, changes become negligible with older age.

Not all values changed in the same manner. Achievement and conformity values did not change at all, not within individuals, nor between generations. Hedonism differed across generations but did not change within individuals over time. Thus, hedonism also seems a stable value; however, over generations change happened. Other values changed between generations, as well as over time. This volatility could mean that societal changes in the values of security, universalism and stimulation may happen faster, not only due to generational change, but also due to adaption of the values within older generations. Further research on the effect of generations is warranted to get a broader insight into the relationship between year of birth (e.g., generations) and age at the time of study with value profiles. Also, as current research is done in a wealthy western (WEIRD) country^53^, further research should corroborate our results in other contexts.

Reflecting back to the question whether differences in value profiles are due to generation, or internal change with age, the answer is nuanced. This study in the Netherlands suggests that value importance in adulthood is mainly a factor of generational differences, although there is some value change with age (mainly in the younger people). However, until this finding replicates across more countries and time periods, the question inevitably remains a somewhat open discussion.

## Method

Our analyses are based on publicly available data collected within the LISS panel (Longitudinal Internet Studies for the Social sciences; www.lissdata.nl), administered by CentERdata (Tilburg University, The Netherlands). The LISS panel is a representative sample of Dutch individuals who participate in monthly Internet surveys. The panel is based on a true probability sample of households drawn from the Dutch population register. Households that could not otherwise participate are provided with a computer and Internet connection. Within this household the same person has filled out the questionnaires. Next to the monthly surveys, a longitudinal survey is fielded in the panel every year, covering a large variety of domains including work, education, income, housing, time use, political views, personality, and values^54^.

### Description of data set

We combined data from the LISS panel from the years 2008 till 2020 (2008, 2009, 2011, 2013, 2014, 2017, 2020). As we intended to analyze intra-individual longitudinal value change over the longest time-period possible within the current panel data, we excluded participants who did not participate in all 7 waves and/or did not fill out the values survey at all these time points. The main reason for including only full responses is comparability across all our analyses and tables. Our final dataset thus includes 7 waves and 1,599 respondents. The respondents included in our study were aged between 16–84 in 2008, and 50.8% were female. The subsample we used from the complete LISS panel was 24% of the original representative dataset. In comparison to the complete LISS panel for which there were human values in 2008 (6,700 respondents) the sample of people who were in the panel for 12 years differed to some extent (see Table 5, and SI 13 Table 14) in supplementary materials). The age difference between people in the panel in 2008 and those who were in our final sample is 46.3 years versus 50.0 years (*t* = −13.59, *p* = 0.000), and average level of education was slightly higher in our sample (higher educated 28.5% in the original sample, versus 31.7% in the final sample X^2^ (1, N = 6700) = 5.9, *p* = 0.016. Furthermore, the number of women was slightly lower in our final sample (50.8%), X^2^(1, N = 6700) = 10.2, *p* = 0.002.

**Table 5:** Description of the dataset and comparison with initial sample (e.g., all respondents who answered the values questions regarding values) in T0.

Dataset	N	Gender (2008)	Age (2008)	Education (2008)
2008	6700	54.3%; women, 45.7% men	46,3 years (range 16 – 95)	High: College/University 28.5%
2008–2020	1599	50.7%; women, 49.3% men	50,0 years (range 16 – 83)	High: College/University 31.4%

### Generations and Schwartz values

For our analyses, we calculated a new variable indicating the generation an individual belongs to: (1) Silent-generation born during 1925–1945, (2) Baby-boomers born during 1946–1964, (3) Generation-X born during 1965–1979, and (4) Millennials born during 1980–1992^36^.

The LISS data includes 36 items that measure values^4^ at each wave. Rokeach and Schwartz values share many commonalities^55^, corroborated by a meta-analysis showing that the circular value structure found with the Schwartz Value Survey (SVS) questionnaire, was also found using the Rokeach Values Survey (RVS)^9^. Previous research has established configural invariance of a similar circular structure in the RVS as in the SVS over 7 countries. The RVS items in the LISS panel have been used before to create values related to the Schwartz framework^10,56^. Using these value items, we created human values that map as closely as4possible onto the Schwartz value framework. We first computed the value scores using the Rokeach items, after which we ipsatized the nine values as advised and commonly done when using Schwartz values^57,58^. See Table 6 for an overview of the used items and the corresponding Cronbach Alphas for each value. Within the supplemental materials we included analyses showing invariance of the value structure across generations (SI 10 Fig. 4).

**Table 6:** Values, scale reliability and scale items.

Value	Cronbach alpha	# Items	Rokeach items
Benevolence	0.781	5	Forgiving, helpful, true friendship, mature love, sincere and truthful
Universalism	0.766	6	Open-minded, a world at peace, equality, wisdom, inner harmony, a world of beauty
Self-direction	0.685	4	Independent, freedom, self-respect, creative
Stimulation	–	1	An exciting life
Hedonism	0.621	2	A comfortable life, pleasure
Achievement	0.698	3	Capable, intellectual, a sense of accomplishment
Power	–	1	Social recognition
Security	0.653	3	Clean, family security, national security
Conformity	0.649	2	Polite, obedient

The data and code for the main analyses can be found at the open science framework (https://osf.io/gp6ef/). In the supplemental material a detailed description of the procedure and items used to construct the Schwartz values is included (SI 3 to 10).

### Data analysis

#### Value change: mean level change and rank-order stability

To assess change, we focused on long-term individual change, and report change in 3 ways: value-profile stability within individuals, followed by rank-order stability and mean level change of each value in the population. We order our results as follows. First, we describe our data, and then assess within-person value-profile stability over time, and whether this is different or similar for different generations using Spearman rank-order correlations.

Second, we focus on between person rank-order stability of the respective values to assess the degree to which human values keep the same relative position in the sample across the 7 time-points. We assess the rank-order-stability across the whole sample, as well as within the 4 distinguished generations, again using Spearman rank-order correlations. The higher the rank-order correlation, the more stable human values are over time. Third, we model value change over time per value using latent growth curve modeling (LGCM)^38,59^. We employed LGCM, an often-used method in developmental research, to provide insight into within individual change over time as well as in the direction of this change. In the analysis our main interest is in differences between generations. For each value, we estimate the following nested models in addition to a base model: (1) a model with random intercept, (2) a model with both random intercept and random slope, (3) random intercept and invariant slope including time- invariant covariates (generation, gender, and education) (4) a model with both random slope and random intercept including time-invariant covariates (generation, gender, and education). To corroborate our analyses, in the supplemental materials (SI 11: table 6.1–9.3) we provide the confidence intervals for these models, plus additional analyses with quadratic and cubic effects for age, and an analysis assessing the effects employing the same LISS sample (N = 2033) containing missing values at some waves.

#### Statistical software

We use R^40^ for our analyses. To calculate descriptive statistics, we use the package psych^60^ and to estimate the latent growth curve models, we employ the R-package lavaan^41^.

### Descriptives

Demographic characteristics and observed scores of value importance at time point 0 (T0; 2008) are reported in Table 7. The sample consists of 1599 individuals with a mean age of 50.0 and 812 (50.7%) females. The scores across the sample are shown in the last row and the other rows show the scores for each of the 4 distinguished generations. At T0 (2008), within the full sample of 1599 individuals, the values benevolence (*M* = 0.683) and self-direction (*M* = 0.417) were considered relatively the most important and both stimulation (*M* = −0.952) and power (*M* = −0.717) the least important. There were some notable differences between the 4 generations in value importance (see F-test in Table 7). Overall, the youngest generation (Millennials) seems most different from the other generations with lower importance attached universalism (0.007), self-direction (0.199), achievement (−0.423) and security (0.062) and higher importance attached to hedonism (0.595) and stimulation (−0.382). The four generations did not differ statistically significantly on benevolence, conformity, and power.

**Table 7:** Demographics, means of the nine human values-types in 2008 (T0), and difference test (ANOVA).

Generation	N	Female (%)	Education (high) (%)	Mean age in 2008 (SD)	Value scores in 2008 (T0)								
					BEN	UNI	SDI	STI	HED	ACH	POW	SEC	CON
Silent	285	43.9	30.5	67.71 (3.98)	0.658	0.385	0.453	−1.232	0.082	−0.231	−0.681	0.544	0.021
Baby-boomers	865	51.3	33.5	53.67 (5.37)	0.710	0.307	0.456	−0.992	0.230	−0.343	−0.732	0.395	−0.03
Gen. X	343	54.2	39.0	37.42 (4.06)	0.672	0.129	0.357	−0.792	0.485	−0.461	−0.749	0.375	−0.016
Millennials	106	52.8	69.8	21.40 (3.39)	0.565	0.007	0.199	−0.382	0.595	−0.423	−0.589	0.062	−0.033
F					2.56	26.35***	8.61***	19.22***	40.31***	7.49***	0.88	16.08***	0.33
Total	1599	50.7	36.6	49.95 (13.1)	0.683	0.263	0.417	−0.952	0.282	−0.354	−0.717	0.395	−0.018

Value-types are measured in 2008 (T0). Education is education in 2020 (T6) to appropriately reflect the level of education of the Millennials. BEN = benevolence, UNI = universalism, SDI = self-direction, STI = stimulation, HED = hedonism, ACH = achievement, POW = power, SEC = security, CON = conformity. Differences between generations are statistically significant for all values except benevolence, power, and conformity.

Significant values are in bold.

*p < 0.05. **p < 0.01. ***p < 0.001.

### Limitations

Our study has some limitations: we use existing panel data within one country, the Netherlands, over a substantial time-period. This limits, to a certain extent, the generalizability of our results, as value change between generations and within people could be specific for this country. Also, as we opted for measuring value change over the longest possible time-period within our respondents (12 years) our final sample was limited to people staying in the panel for this period, which could have led to deviations from the nationally representative sample. Comparing generations also led to an uneven amount of people in each generation, with smaller groups for the youngest and oldest groups. Although we cross validated our measure with other measures, as we used the RVS items to construct Schwartz values, this could also have influenced some of the results, as there were some differences in items and wording between the original scale and our selection of Rokeach value items.

### Institutional review board statement

Ethical review and approval were waived for this study because only publicly available data were used.

### Informed consent statement

Informed consent was obtained from all subjects involved in the study. A detailed description of the informed consent can be found at the website of the LISS Panel (https://www.dataarchive.lissdata.nl/. Accessed on December 16, 2021).

## Supplementary Information

Supplementary Information.

## Data Availability

Individual Data for the paper is publicly available at the website of the LISS Panel (https://www.dataarchive.lissdata.nl/, (accessed on December 16, 2021)). The specific dataset and relevant coding is provided at https://osf.io/gp6ef/.
