# Author Correction: Individual and generational value change in an adult population, a 12-year longitudinal panel study

**DOI:** 10.1038/s41598-023-28887-4

**Published:** 2023-02-02

**Authors:** Ingmar Leijen, Hester van Herk, Anat Bardi

**Affiliations:** 1 School of Business and Economics, Vrije Universiteit Amsterdam, Amsterdam, The Netherlands; 2 Royal Holloway University of London, Egham, UK

Correction to: *Scientific Reports* 10.1038/s41598-022-22862-1, published online 25 October 2022

The original version of this Article contained an error in Reference 46, which was incorrectly given as:

Daniel, E. Value development during adolescence: Dimensions of change and stability. *J. Pers.* 87, 620 (2019).

The correct reference is listed below:

Daniel, E., & Benish‐Weisman, M. Value development during adolescence: Dimensions of change and stability. *J. Pers.*, **87**(3), 620–632 (2019).

The original Article has been corrected.

