# Supplementary material for: Individual and generational value change in an adult population, a 12-year longitudinal panel study
Source: Sci Rep. 2022 Oct 25;12:17844. doi: 10.1038/s41598-022-22862-1 (PMC9596497; doi:10.1038/s41598-022-22862-1)
Supplement: Supplementary file 1 — Supplementary Information. [file 41598_2022_22862_MOESM1_ESM.docx]

**Supplementary materials**

SI 1: Fit statistics LCGM models (SI Table 1).

SI 2: Value Profile Stability Graph (SI Fig. 1).

SI 3: Background information construction of the human values construct (SI Fig. 2).

SI 4: List of items and translation to Schwartz human values (SI Table 2).

SI 5: Appropriateness of the value measurement.

SI 6: Comparison Schwartz values European Social Survey and LISS panel (SI Table 3, SI Fig. 3).

SI 7: Correlations between values (SI Table 4).

SI 8: Correlation LISS panel WVS Schwartz Values and Rokeach/Schwartz Values (SI Table 5).

SI 9: Period of data collection, gender, and education.

SI 10: Invariance of the value configuration across generations (SI Fig. 4).

SI 11: LCGM models with p values and confidence intervals.

- SI Tables 6.1-6.3: Model in the paper including confidence intervals (N=1,599).
- SI Tables 7.1-7.3: Model with only age effects (linear, squared, and cubic) (N=1,599).
- SI Tables 8.1-8.3: Model as in SI tables 6.1-6.3 with age effects within generations added (N=1,599).
- SI Tables 9.1-9.3: Model as in SI tables 6.1-6.3 based on the same LISS sample with missing observations in one or more of the in between waves (N=2,033).

SI 12: T-test comparing within and between cohorts.

- SI Table 10, 11 (N=1,599)
- SI Table 12, 13 (N=2,033))

SI 13: Sample composition (SI Table 14).

SI 14: Reference list supplementary materials.

**SI 1: Fit statistics Latent Curve Growth Models (LCGM).**

SI Table 1. Fit statistics of the growth models, the last model (df=59) refers to the model reported in the article, Table 4 main text.

|  |  | Intercept  and slope*  (df = 71) | Intercept + slope +  time-invariant covariates  intercept (df = 65) | Intercept + slope +  time-invariant covariates  intercept and slope (df = 59) |
| --- | --- | --- | --- | --- |
| Benevolence | Chi-square | 224.157 | 156.668 | **135.956** |
|  | Chi-square delta |  | 67.488 | 20.712 |
|  | RMSEA | 0.037 | 0.03 | 0.029 |
|  | SRMR | 0.047 | 0.024 | 0.022 |
|  | CFI | 0.971 | 0.982 | 0.985 |
| Universalism | Chi-square | 426.416 | 185.710 | 145.537 |
|  | Chi-square delta |  | 240.706 | 40.173 |
|  | RMSEA | 0.056 | 0.034 | 0.03 |
|  | SRMR | 0.077 | 0.029 | 0.025 |
|  | CFI | 0.944 | 0.981 | 0.986 |
| Self-direction | Chi-square | 218.629 | 125.426 | 87.927 |
|  | Chi-square delta |  | 93.230 | 37.499 |
|  | RMSEA | 0.036 | 0.024 | 0.018 |
|  | SRMR | 0.049 | 0.019 | 0.016 |
|  | CFI | 0.974 | 0.989 | 0.995 |
| Stimulation | Chi-square | 291.346 | 153.574 | 97.285 |
|  | Chi-square delta |  | 137.772 | 56.289 |
|  | RMSEA | 0.044 | 0.029 | 0.02 |
|  | SRMR | 0.058 | 0.024 | 0.019 |
|  | CFI | 0.96 | 0.984 | 0.993 |
| Hedonism | Chi-square | 421.861 | 139.227 | 126.615 |
|  | Chi-square delta |  | 282.634 | 12.61 |
|  | RMSEA | 0.056 | 0.027 | 0.027 |
|  | SRMR | 0.077 | 0.028 | 0.027 |
|  | CFI | 0.923 | 0.984 | 0.985 |
| Achievement | Chi-square | 315.538 | 118.122 | 110.971 |
|  | Chi-square delta |  | 197.417 | 7.33 NS |
|  | RMSEA | 0.046 | 0.023 | 0.023 |
|  | SRMR | 0.066 | 0.021 | 0.02 |
|  | CFI | 0.944 | 0.988 | 0.988 |
| Power | Chi-square delta | 208.913 | 158.779 | 122.992 |
|  | Chi-square |  | 50.134 | 35.787 |
|  | RMSEA | 0.035 | 0.03 | 0.026 |
|  | SRMR | 0.042 | 0.026 | 0.023 |
|  | CFI | 0.963 | 0.975 | 0.983 |
| Security | Chi-square | 396.257 | 197.139 | 144.182 |
|  | Chi-square delta |  | 199.118 | 52.957 |
|  | RMSEA | 0.054 | 0.036 | 0.03 |
|  | SRMR | 0.083 | 0.032 | 0.028 |
|  | CFI | 0.945 | 0.978 | 0.976 |
| Conformity | Chi-square | 263.101 | 142.788 | 134.792 |
|  | Chi-square delta |  | 120.313 | 8.000 NS |
|  | RMSEA | 0.041 | 0.027 | 0.028 |
|  | SRMR | 0.061 | 0.024 | 0.023 |
|  | CFI | 0.959 | 0.983 | 0.984 |

*All coefficients are significant with P < .05, except when indicated NS. * This is the model in which all coefficients except slope and intercept are set to zero to allow comparison between the two other models.*

**SI 2: Value Profile Stability Plot.**

SI Fig. 1. Visualization of the value profile stability per generation.

**
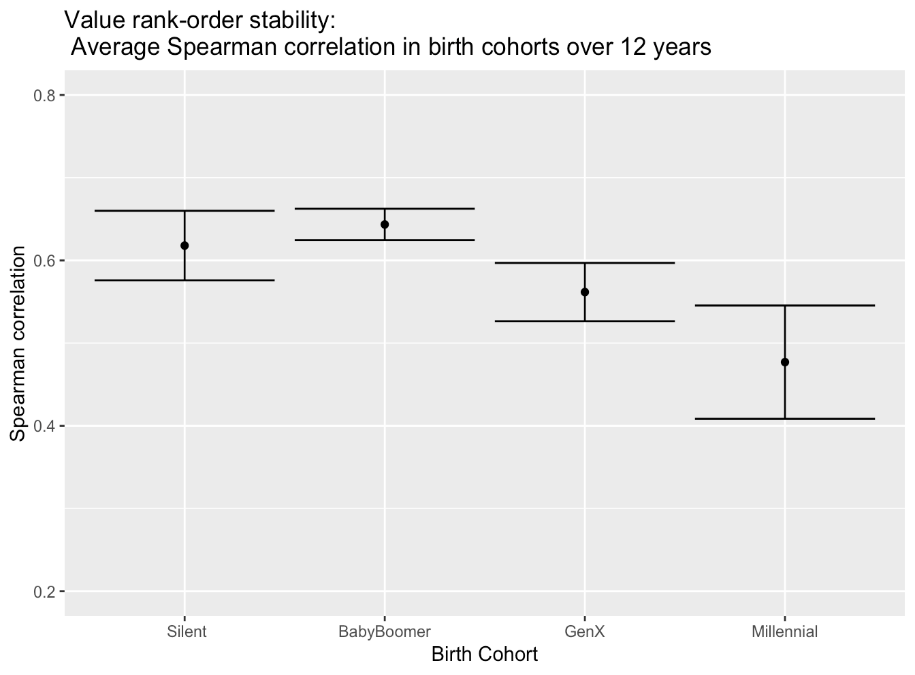
**

As can be seen from the graph the correlation between value profiles in 2008 and 2020 became slightly lower again for the Silent Generation. Also, an increase in variance is visible, making the difference not significant. Lowest correlation between value profiles is found in the youngest generations (Millennials) Also the variance within the group is highest for the millennials, indicating that within the youngest group there is the most variance in stability. The most stable group in value profile is the baby-boom generation: Both a high correlation as well as a low variance indicating less difference within the group in value profile stability.**SI 3: Background information construction of the human values construct.**

SI Fig. 2. Plot of ipsatized scores for the Schwartz values (PVQ11) and the values constructed with Rokeach Items in 2011 and 2013 (all in LISS panel).

We used the Schwartz values conceptualization from his 1992 article and compared this with the items from the original Rokeach values questionnaire. For 21 items we found an exactly equivalent item, and for an additional 6 items we found items that resembled very closely to another item within the Schwartz scale. There were no items that could be attributed to the value of tradition. However, we found equivalent items measuring conformity, which is closely related to the value of tradition within the value circumplex of Schwartz’s theory. Thus, we constructed a set of 9 values closely resembling 9 of the 10 values from the value circumplex proposed by Schwartz. In SI Fig. 2 we visualize the resemblance, and we also add a correlation table (SI Table 5)

We also included some of the items of the Rokeach scale that were almost equivalent in wording and meaning with the Schwartz items to improve construct validity. We dismissed all items that did not have a clear overlap with a corresponding Schwartz item. Item 104 (courageous) showed overlap but was not included as the Dutch translation was clearly confusing and multi-interpretable. The items that were added to the scales that were not literally the same are marked with an * in SI Table 2. In the corresponding column, the Schwartz item that we considered equivalent is mentioned. There are 2 values that are measured with only one item: Power and Stimulation. For the other 7 values there are multiple items. The multi-item scales show a reliability of minimum .622 (Hedonism) up to .810 (benevolence). Scale reliability has been calculated with the raw item scores of the full sample in 2008.

We acknowledge that the items are scored slightly different from the proposed procedure by Shalom H. Schwartz (1992). The Rokeach survey uses a scale of 1 (extremely unimportant) to 7 (extremely important), while the Schwartz survey uses a scale of -1 (opposing my values) to 7. We assume that the effect of the different scaling will be limited, as research has shown that people will only sporadically choose the -1 item, leaving comparability with the original Schwartz values sufficiently high (Vauclair, Hanke, Fischer, & Fontaine, 2011). Furthermore, we recognize there has been considerable development in the way the Schwartz value framework is measured in current research (Lee, Soutar, & Louviere, 2008). However, Rokeach values have been used before as a proxy for the Schwartz values framework.

**SI 4: List of items and translation to Schwartz human values.**

*SI Table 2. List of items and translation to Schwartz human values.*

| Item | Dutch item | English item | Schwartz alternative | Value |
| --- | --- | --- | --- | --- |
| A107 | competent | Capable |  | Achievement |
| A113 | intellectueel* | Intellectual | intelligent | Achievement |
| A127 | prestatie* | a sense of accomplishment | successful | Achievement |
| A100 | verantwoordelijk | Responsible |  | Benevolence |
| A102 | vergevingsgezind | Forgiving |  | Benevolence |
| A105 | behulpzaam | Helpful |  | Benevolence |
| A126 | ware vriendschap | true friendship |  | Benevolence |
| A130 | liefde en seksualiteit | mature love |  | Benevolence |
| A099 | eerlijk, oprecht* | sincere and truthful | honest | Benevolence |
| A112 | beleefd* | Polite | politeness | Conformity |
| A114 | gehoorzaam | Obedient |  | Conformity |
| A129 | comfortabel leven | a comfortable life | enjoying life | Hedonism |
| A132 | plezier | pleasure |  | Hedonism |
| A133 | erkenning, status | social recognition |  | Power |
| A108 | netjes | clean |  | Security |
| A118 | het gezin | family security |  | Security |
| A124 | nationale veiligheid | national security |  | Security |
| A110 | onafhankelijk | independent |  | Self-direction |
| A119 | vrijheid | freedom |  | Self-direction |
| A116 | creativiteit* | creative | creativity | Self-direction |
| A121 | zelfrespect | self-respect |  | Self-direction |
| A134 | opwindend leven | an exciting life |  | Stimulation |
| A103 | open* | open-minded | broadminded | Universalism |
| A117 | wereldvrede | a world at peace |  | Universalism |
| A120 | gelijkheid | equality |  | Universalism |
| A123 | wijsheid | wisdom |  | Universalism |
| A128 | innerlijke harmonie | inner harmony |  | Universalism |
| A131 | schoonheid | a world of beauty |  | Universalism |

*Items that were literally the same have been added to the value type as indicated. Items with a closely resembling meaning have been added accordingly. Dutch items as used in questionnaire are indicated in the first row. “*” means that the alternative items have been included. Values that had no literal or closely resembling equivalent have not been included.*

SI 5: Appropriateness of the value measurement.

To assess the similarity between Rokeach and Schwartz we used the original Schwartz value items as used in the WVS ([www.worldvaluessurvey.org](http://www.worldvaluessurvey.org)) that were measured in in 2012 in the same panel; in SI Fig. 2 we have combined the means for the original Schwartz values with the means for the values we constructed from the Rokeach items from 2011 and 2013. As can be seen in this figure there are some differences, but overall, there is strong similarity with a correlation of .91 and .90 with 2011 and 2013 respectively (N=18). We also correlated the subsample of around 700 respondents for which we had the WVS item scores from 2012 with the 2011 values per value item, which showed that similar positive and negative correlations were present for the WVS 2012 and the Rokeach 2011 values. (See SI Table 5)

To further determine the appropriateness of the Rokeach items to be used as a proxy for the Schwartz values, we also assessed whether our measure of the values was comparable with the Schwartz PVQ scale (www.worldvaluessurvey.org), which was administrated to part of our LISS sample in 2012 (N=743). We calculated the correlation of the ipsatized means of the 9 values (from the 2013 wave) with the ipsatized means of the 9 Schwartz values of the 2012 wave and found a strong correlation of .86 over all values, indicating similarity (SI Fig. 3).

Also, as our sample consists of a random sample of the Dutch population, we can compare the average value priority scores with known national scores on the Schwartz Values within the Netherlands^1^. We investigated European Social Survey waves 2008, 201, 2012 and 2014 to link them to the same years of the data collection of the LISS panel. In these data we found similar value priorities within the Dutch sample, adding to the appropriateness of our measure as a proxy for the Schwartz values. A similar graph as with the WVS items in the LISS panel, but now comparing our values with another nationally representative sample is provided below. (See SI Table 3 and SI Fig. 3).

To further assess the validity of our values, we correlated individual human values in 2008 with age, gender, and level of education (see **S7: Correlations between values**). The 9 values seem to have sufficient similarity with the nine corresponding Schwartz values. We found high similarity with Schwartz values measured in ESS data and as well as in the correlations with common indicators like gender, education, and age.

Note that a minimum value-profile stability of -1 was found (see Table 1 main text), indicating that the sample also included people whose value-profile changed substantially and even became negative. Although theoretically such a change may be possible, it is not likely, and it is possible that this reversal is due to people erroneously reading the answering options in the reverse directions either in 2008 or 2020. Nevertheless, as we cannot be sure if this is a mistake or a real value change, we retained these responses in the final dataset.

**SI 6: Comparison Schwartz values European Social Survey and LISS panel.**

*SI* *Table 3. Means for Ipsatized Schwartz Values of the Dutch sample from the European Social Survey (combined wave 4 to 8) and LISS panel (wave T0 (2008) to T6 (2020).*

|  | Ben | Uni | Sdi | Sti | Hed | Ach | Pow | Sec | Con |
| --- | --- | --- | --- | --- | --- | --- | --- | --- | --- |
| LISS: | 0.687 | 0.30 | 0.44 | -1.02 | 0.29 | -0.35 | -0.80 | 0.49 | -0.04 |
| ESS: | 0.65 | 0.54 | 0.53 | -0.53 | 0.01 | -0.50 | -1.09 | 0.03 | -0.16 |

SI Fig. 3. Mean Values for Ipsatized Schwartz Values of the European Social Survey (wave 4-8) and LISS panel human values (T0 (2008)-T6 (2020)). Values in SI Table 3 correspond with the graph below.

**SI 7: Correlations between values.**

SI Table 4. Correlations between values in our sample in 2008 (N=1,599)

|  | Mean | SD |  | 1 | 2 | 3 | 4 | 5 | 6 | 7 | 8 | 9 |
| --- | --- | --- | --- | --- | --- | --- | --- | --- | --- | --- | --- | --- |
| 1. benevolence | 0.684 | 0.545 |  | 1 | .406*** | .295*** | -.424*** | -.013 | -.230*** | -.533*** | .194*** | .148*** |
| 2. universalism | 0.265 | 0.500 |  | .406*** | 1 | .352*** | -.351*** | -.122*** | -.116*** | -.392*** | .142 | -.118*** |
| 3. self-direction | 0.420 | 0.582 |  | .295*** | .354*** | 1 | -.294*** | -.070** | -.018 | -.396*** | -.041 | -.136*** |
| 4. stimulation | -0.954 | 1.101 |  | -.424*** | -.351*** | -.294*** | 1 | .031 | -.144*** | .140*** | -.464 | -.393*** |
| 5. hedonism | 0.282 | 0.578 |  | -.013 | -.122*** | -.070** | .031 | 1 | -.226*** | -.137*** | -.138 | -.186*** |
| 6. achievement | -0.355 | 0.639 |  | -.230*** | -.116*** | -.018 | -.144*** | -.226*** | 1 | .026 | -.164 | -.094*** |
| 7. power | -0.720 | 1.006 |  | -.533*** | -.392*** | -.396*** | .140*** | -.137*** | .026 | 1 | -.281 | -.270*** |
| 8. security | 0.395 | 0.617 |  | .194*** | .142*** | -.041 | -.464*** | -.138*** | -.164*** | -.281*** | 1 | .257*** |
| 9. conformity | -0.018 | 0.760 |  | .148*** | -.118*** | -.136*** | -.393*** | -.186*** | -.094*** | -.270*** | .257 | 1 |

*P < 0.001 level (2-tailed) *** P < 0.01 level (2-tailed), * P < 0.05 level (2-tailed), N=1599*

**SI 8: Correlation LISS panel WVS Schwartz Values and Rokeach/Schwartz Values.**

| *SI Table 5. Correlations between LISS panel WVS Schwartz Values in 2012 and Rokeach/Schwartz Values in 2011* |
| --- |

|  | Ben | Uni | Sdi | Sti | Hed | Ach | Pow | Sec | Con |
| --- | --- | --- | --- | --- | --- | --- | --- | --- | --- |
| Ben (WVS) ^1^ | .192** | .132** | .069 | -.211** | -.162** | -.064 | -.130** | .080* | -.009 |
| Uni (WVS) | .064 | .212** | .052 | -.196** | -.113** | -.093* | -.177** | .123** | -.008 |
| Sdi (WVS) | -.008 | .010 | .100** | .050 | .004 | .021 | -.056 | -.170** | -.150** |
| Sti (WVS) | -.115** | -.047 | -.018 | .336** | .067 | .097** | .128** | -.199** | -.136** |
| Hed (WVS) | .000 | -.035 | .051 | .136** | .264** | -.030 | .022 | -.142** | -.050 |
| Ach (WVS) | -.162** | -.177** | -.084* | .220** | .075* | .147** | .256** | -.171** | -.058 |
| Pow (WVS) | -.041 | -.085* | .022 | .210** | .166** | .095* | .147** | -.091* | -.081* |
| Sec (WVS) | .013 | .022 | -.031 | -.187** | -.034 | -.060 | -.053 | .188** | .105** |
| Con (WVS) | .056 | -.028 | -.037 | -.173** | -.022 | -.048 | -.057 | .197** | .209** |

| *** Correlation is significant at the 0.01 level (2-tailed), * Correlation is significant at the 0.05 level (2-tailed).* |
| --- |

*^1^(WVS) indicates: values measured using WVS Schwartz values scale in 2012. Horizontally values measured using Rokeach items in 2011. N differed slightly per comparison; Ben = 714, Uni = 708, Sdi = 699, Sti, Hed = 710, Ach, Pow = 711, Sec = 712, Con = 706. The WVS version of the Schwartz values is also an approximation of the original Schwartz Values, as there are only 11 items measuring the 10 human values. Unfortunately, the official Schwartz PVQ or SVS value scale have not been measured in the LISS panel to date. Correlations of WVS and Rokeach value approximations are following the common pattern as predicted with theory^2^.*

SI 9: Period of data collection, gender, and education.

**Gender:** Woman (0), Man (1).

**Education**:

Education was coded as a binomial variable; we coded all educational levels up to high school or vocational education low (0) and higher vocational/university as high. We used CBS coding to obtain this variable (the Central Population Statistics Agency of the Netherlands). For the dataset we used the educational level in 2017.

Period of data collection of variables per period in the LISS panel:

- 2008: May, August
- 2009 May, June
- 2010: May, June
- 2011: May, June
- 2012: May, June
- 2013: May, June
- 2014: November, December
- 2015: November, December
- 2017: May, June
- 2020: June

**SI 10: Invariance of the value configuration across generations.**

To assess invariance of the values structure across generations, we first determined the structure of our values in the respective generations with multi-dimensional scaling analysis using SMACOF in R^3,4^. Next, to assess the invariance of the values configurations between the four generations we used the Procrustean rotation and calculated the congruence coefficient as recommended^5,6^ As the reference configuration we used the configuration of the Silent Generation. The results can be seen in SI Fig. 4. The congruence coefficients of the configurations for the Baby Boomers, Generation X and the Millennials are .992, .989, and .982 respectively. These numbers indicate a good correspondence between the respective configurations and invariance of the values structure across generations.

*SI Fig. 4. Congruence between the MDS configurations of the Silent Generation and Baby Boomers, Generation X and Millennials. The target configuration is the Silent Generation.*

|  |  |  |
| --- | --- | --- |

**SI 11: LCGM models with p values and confidence intervals.**

SI Table 6.1. Results of the Latent Growth Curve Models per value using the SEM approach (N=1,599). For each value the coefficients related to the latent intercept are described, these coefficients show first the mean level of the value followed by coefficeints related to diffrences in the mean. Next, the latent slope is given which indicates change over the 12 year period in the study, followed by the coefficients related to the change. For each value the same model is shown in this table to allow comparison; for some values (e.g., for Hedonism) the best model fit is a more parsimonious model (see SI Table 1).

|  | Benevolence | | | | Universalism | | | | Self-direction | | | |
| --- | --- | --- | --- | --- | --- | --- | --- | --- | --- | --- | --- | --- |
|  | Coef. | P value | CI lower | CI Higher | Coef. | P value | CI lower | CI Higher | Coef. | P value | CI lower | CI Higher |
| Latent Intercept | 0.767 | 0.000 | 0.711 | 0.826 | 0.485 | 0.000 | 0.435 | 0.534 | 0.434 | 0.000 | 0.379 | 0.493 |
| Gender (male=1) | -0.185 | 0.000 | -0.234 | -0.143 | -0.200 | 0.000 | -0.240 | -0.161 | -0.062 | 0.010 | -0.113 | -0.015 |
| Education (high = 1) | 0.050 | 0.041 | 0.000 | 0.098 | 0.091 | 0.000 | 0.046 | 0.135 | 0.174 | 0.000 | 0.122 | 0.228 |
| Age^**^ | 0.002 | 0.433 | -0.003 | 0.006 | 0.011 | 0.000 | 0.007 | 0.015 | 0.002 | 0.339 | -0.003 | 0.007 |
| Baby-boomers^*^ | 0.014 | 0.635 | -0.044 | 0.072 | -0.108 | 0.000 | -0.158 | -0.055 | -0.014 | 0.627 | -0.071 | 0.040 |
| Generation-X | -0.054 | 0.145 | -0.121 | 0.019 | -0.323 | 0.000 | -0.387 | -0.262 | -0.107 | 0.003 | -0.179 | -0.034 |
| Millennials | -0.138 | 0.006 | -0.232 | -0.034 | -0.498 | 0.000 | -0.585 | -0.406 | -0.393 | 0.000 | -0.500 | -0.294 |
| Latent Slope | -0.005 | 0.072 | -0.010 | 0.000 | -0.001 | 0.532 | -0.006 | 0.003 | -0.005 | 0.046 | -0.010 | 0.000 |
| Gender (male=1) | 0.004 | 0.043 | 0.000 | 0.008 | 0.002 | 0.306 | -0.002 | 0.005 | 0.000 | 0.939 | -0.004 | 0.004 |
| Education (high = 1) | -0.001 | 0.739 | -0.004 | 0.003 | 0.002 | 0.201 | -0.001 | 0.006 | 0.001 | 0.758 | -0.004 | 0.005 |
| Age^2^ | 0.000 | 0.134 | -0.001 | 0.000 | 0.000 | 0.022 | -0.001 | 0.000 | -0.001 | 0.008 | -0.001 | 0.000 |
| Baby-boomers | 0.003 | 0.327 | -0.003 | 0.008 | 0.006 | 0.010 | 0.001 | 0.010 | 0.009 | 0.000 | 0.004 | 0.014 |
| Generation-X | 0.006 | 0.064 | 0.000 | 0.012 | 0.011 | 0.000 | 0.005 | 0.016 | 0.014 | 0.000 | 0.007 | 0.020 |
| Millennials | 0.017 | 0.001 | 0.007 | 0.026 | 0.018 | 0.000 | 0.009 | 0.028 | 0.022 | 0.000 | 0.012 | 0.033 |

*^*^Silent generation is the reference category. ^**^Age is age-centered on the group mean of each generation to avoid multicollinearity with the birth cohort variable.*

SI Table 6.2. Results of the Latent Growth Curve Models per value using the SEM approach (N=1599). For each value the coefficients related to the latent intercept are described, these coefficients show first the mean level of the value followed by coefficeints related to diffrences in the mean. Next, the latent slope is given which indicates change over the 12 year period in the study, followed by the coefficients related to the change. For each value the same model is shown in this table to allow comparison; for some values (e.g., for Hedonism) the best model fit is a more parsimonious model (see SI Table 1).

|  | Stimulation | | | | Hedonism | | | | Achievement | | | |
| --- | --- | --- | --- | --- | --- | --- | --- | --- | --- | --- | --- | --- |
|  | Coef. | P value | CI lower | CI Higher | Coef. | P value | CI lower | CI Higher | Coef. | P value | CI lower | CI Higher |
| Intercept mean | -1.392 | 0.000 | -1.505 | -1.265 | 0.050 | 0.090 | -0.005 | 0.110 | -0.327 | 0.000 | -0.392 | -0.265 |
| Gender (male=1) | 0.393 | 0.000 | 0.309 | 0.479 | 0.014 | 0.554 | -0.032 | 0.062 | 0.091 | 0.000 | 0.046 | 0.140 |
| Education (high = 1) | -0.144 | 0.002 | -0.237 | -0.053 | -0.028 | 0.251 | -0.077 | 0.020 | 0.261 | 0.000 | 0.207 | 0.313 |
| Age^**^ | -0.023 | 0.000 | -0.031 | -0.014 | -0.015 | 0.000 | -0.019 | -0.010 | 0.002 | 0.358 | -0.003 | 0.007 |
| Baby-boomers^*^ | 0.279 | 0.000 | 0.168 | 0.396 | 0.192 | 0.000 | 0.129 | 0.253 | -0.161 | 0.000 | -0.226 | -0.093 |
| Generation-X | 0.517 | 0.000 | 0.373 | 0.664 | 0.435 | 0.000 | 0.356 | 0.511 | -0.306 | 0.000 | -0.384 | -0.229 |
| Millennials | 0.977 | 0.000 | 0.769 | 1.188 | 0.554 | 0.000 | 0.443 | 0.668 | -0.270 | 0.000 | -0.381 | -0.153 |
| Slope mean | 0.008 | 0.141 | -0.002 | 0.019 | 0.001 | 0.668 | -0.004 | 0.007 | -0.003 | 0.251 | -0.009 | 0.002 |
| Gender (male=1) | -0.007 | 0.082 | -0.015 | 0.001 | -0.006 | 0.008 | -0.011 | -0.002 | 0.003 | 0.148 | -0.001 | 0.008 |
| Education (high = 1) | 0.001 | 0.715 | -0.007 | 0.009 | 0.003 | 0.266 | -0.002 | 0.007 | -0.005 | 0.057 | -0.009 | 0.000 |
| Age^2^ | 0.001 | 0.005 | 0.000 | 0.002 | 0.000 | 0.842 | 0.000 | 0.000 | 0.000 | 0.344 | 0.000 | 0.001 |
| Baby-boomers | -0.022 | 0.000 | -0.032 | -0.011 | 0.003 | 0.333 | -0.003 | 0.008 | 0.002 | 0.578 | -0.004 | 0.007 |
| Generation-X | -0.033 | 0.000 | -0.046 | -0.022 | 0.004 | 0.302 | -0.003 | 0.011 | 0.005 | 0.181 | -0.002 | 0.012 |
| Millennials | -0.054 | 0.000 | -0.075 | -0.033 | 0.009 | 0.174 | -0.005 | 0.021 | 0.005 | 0.404 | -0.007 | 0.018 |

*^*^Silent-generation is the reference category. ^**^Age is age centered on the group mean of each generation*

SI Table 6.3. Results of the Latent Growth Curve Models per value using the SEM approach (N=1599). For each value the coefficients related to the latent intercept are described, these coefficients show first the mean level of the value followed by coefficeints related to diffrences in the mean. Next, the latent slope is given which indicates change over the 12 year period in the study, followed by the coefficients related to the change. For each value the same model is shown in this table to allow comparison; for some values (e.g., for Hedonism) the best model fit is a more parsimonious model (see SI Table 1).

|  | Power | | | | Security | | | | Conformity | | | |
| --- | --- | --- | --- | --- | --- | --- | --- | --- | --- | --- | --- | --- |
|  | Coef. | P value | CI lower | CI Higher | Coef. | P value | CI lower | CI Higher | Coef. | P value | CI lower | CI Higher |
| Intercept mean | -0.924 | 0.000 | -1.025 | -0.808 | 0.744 | 0.000 | 0.685 | 0.805 | 0.163 | 0.000 | 0.086 | 0.243 |
| Gender (male=1) | 0.263 | 0.000 | 0.177 | 0.346 | -0.188 | 0.000 | -0.238 | -0.138 | -0.125 | 0.000 | -0.187 | -0.060 |
| Education (high = 1) | 0.093 | 0.029 | 0.009 | 0.178 | -0.247 | 0.000 | -0.305 | -0.191 | -0.250 | 0.000 | -0.312 | -0.184 |
| Age^**^ | -0.001 | 0.783 | -0.010 | 0.007 | 0.012 | 0.000 | 0.007 | 0.017 | 0.009 | 0.006 | 0.003 | 0.015 |
| Baby-boomers^*^ | 0.025 | 0.627 | -0.077 | 0.126 | -0.155 | 0.000 | -0.221 | -0.089 | -0.073 | 0.061 | -0.151 | 0.001 |
| Generation-X | 0.014 | 0.826 | -0.121 | 0.133 | -0.199 | 0.000 | -0.286 | -0.122 | 0.023 | 0.633 | -0.075 | 0.117 |
| Millennials | 0.201 | 0.028 | 0.010 | 0.380 | -0.433 | 0.000 | -0.567 | -0.304 | 0.001 | 0.993 | -0.147 | 0.137 |
| Slope mean | 0.007 | 0.124 | -0.002 | 0.017 | 0.003 | 0.294 | -0.002 | 0.008 | -0.005 | 0.144 | -0.012 | 0.002 |
| Gender (male=1) | -0.007 | 0.089 | -0.016 | 0.001 | 0.005 | 0.043 | 0.000 | 0.010 | 0.006 | 0.040 | 0.001 | 0.011 |
| Education (high = 1) | -0.007 | 0.123 | -0.015 | 0.002 | 0.006 | 0.006 | 0.002 | 0.011 | -0.002 | 0.519 | -0.007 | 0.004 |
| Age^**^ | 0.000 | 0.279 | 0.000 | 0.001 | 0.000 | 0.047 | -0.001 | 0.000 | 0.000 | 0.818 | -0.001 | 0.001 |
| Baby-boomers ^*^ | -0.015 | 0.003 | -0.024 | -0.005 | 0.012 | 0.000 | 0.007 | 0.018 | 0.002 | 0.551 | -0.005 | 0.009 |
| Generation-X | -0.024 | 0.000 | -0.037 | -0.012 | 0.011 | 0.002 | 0.004 | 0.018 | 0.007 | 0.101 | -0.002 | 0.015 |
| Millennials | -0.043 | 0.000 | -0.064 | -0.024 | 0.027 | 0.000 | 0.014 | 0.040 | -0.001 | 0.861 | -0.016 | 0.014 |

*^*^Silent-generation is the reference category. ^**^Age is age centered on the group mean of each generation*

SI Table 7.1. Results of the Latent Growth Curve Models per value using the SEM approach (N=1599)^7^ with age, age squared, and age cubic as explanatory variables and no cohort effects.^7^ In the analysis age was divided by 10 to give the variabels a comparable scale. A change of 1 unit in the coefficient here refers to a change in 10 years of age.

|  | Benevolence | | | | Universalism | | | | Self-direction | | | |
| --- | --- | --- | --- | --- | --- | --- | --- | --- | --- | --- | --- | --- |
|  | Coef. | P value | CI lower | CI Higher | Coef. | P value | CI lower | CI Higher | Coef. | P value | CI lower | CI Higher |
| Intercept mean | 0.775 | 0.000 | 0.734 | 0.814 | 0.337 | 0.000 | 0.307 | 0.371 | 0.410 | 0.000 | 0.371 | 0.451 |
| Gender (male=1) | -0.186 | 0.000 | -0.231 | -0.140 | -0.202 | 0.000 | -0.240 | -0.162 | -0.063 | 0.009 | -0.111 | -0.016 |
| Education (high = 1) | 0.051 | 0.052 | 0.003 | 0.105 | 0.091 | 0.000 | 0.048 | 0.136 | 0.173 | 0.000 | 0.123 | 0.227 |
| Age linear ^*^ | 0.029 | 0.075 | -0.003 | 0.062 | 0.133 | 0.000 | 0.104 | 0.164 | 0.025 | 0.112 | -0.006 | 0.055 |
| Age Squared^*^ | -0.014 | 0.022 | -0.025 | -0.001 | -0.010 | 0.053 | -0.022 | 0.000 | -0.014 | 0.017 | -0.026 | -0.002 |
| Age Cubic^*^ | -0.002 | 0.484 | -0.008 | 0.004 | -0.006 | 0.041 | -0.012 | 0.000 | 0.006 | 0.043 | 0.000 | 0.011 |
| Slope mean | -0.001 | 0.584 | -0.004 | 0.002 | 0.006 | 0.000 | 0.003 | 0.008 | 0.005 | 0.006 | 0.001 | 0.008 |
| Gender (male=1) | 0.004 | 0.050 | 0.000 | 0.008 | 0.002 | 0.297 | -0.002 | 0.005 | 0.000 | 0.968 | -0.004 | 0.004 |
| Education (high = 1) | -0.001 | 0.803 | -0.004 | 0.004 | 0.003 | 0.161 | -0.001 | 0.006 | 0.001 | 0.723 | -0.003 | 0.005 |
| Age linear^*^ | 0.000 | 0.775 | -0.003 | 0.002 | -0.003 | 0.011 | -0.006 | -0.001 | -0.004 | 0.012 | -0.006 | -0.001 |
| Age Squared^*^ | 0.000 | 0.433 | -0.001 | 0.001 | 0.000 | 0.338 | -0.001 | 0.000 | -0.001 | 0.236 | -0.002 | 0.000 |
| Age Cubic^*^ | -0.001 | 0.033 | -0.001 | 0.000 | 0.000 | 0.394 | -0.001 | 0.000 | 0.000 | 0.345 | -0.001 | 0.000 |

*^*^ Age is grand-mean centered in the sample in these analyses to avoid multicollinearity between the age variables.*

SI Table 7.2*.* Results of the Latent Growth Curve Models per value using the SEM approach (N=1599)^7^ with age, age squared, and age cubic as explanatory variables and no cohort effects.^7^ In the analysis age was divided by 10 to give the variabels a comparable scale. A change of 1 unit in the coefficient here refers to a change in 10 years of age.

|  | Stimulation | | | | Hedonism | | | | Achievement | | | |
| --- | --- | --- | --- | --- | --- | --- | --- | --- | --- | --- | --- | --- |
|  | Coef. | P value | CI lower | CI Higher | Coef. | P value | CI lower | CI Higher | Coef. | P value | CI lower | CI Higher |
| Intercept mean | -1.09 | .000 | -1.168 | -1.019 | 0.295 | 0.000 | 0.252 | 0.331 | -0.523 | 0.000 | -0.561 | -0.485 |
| Gender (male=1) | 0.395 | .000 | 0.308 | 0.483 | 0.014 | 0.547 | -0.029 | 0.062 | 0.090 | 0.000 | 0.039 | 0.138 |
| Education (high = 1) | -0.136 | .003 | -0.227 | -0.039 | -0.028 | 0.260 | -0.077 | 0.018 | 0.261 | 0.000 | 0.208 | 0.315 |
| Age linear ^*^ | -0.106 | .001 | -0.168 | -0.040 | -0.159 | 0.000 | -0.192 | -0.126 | 0.092 | 0.000 | 0.059 | 0.124 |
| Age Squared^*^ | -0.019 | .187 | -0.047 | 0.010 | -0.003 | 0.660 | -0.014 | 0.010 | 0.013 | 0.055 | 0.000 | 0.026 |
| Age Cubic^*^ | -0.020 | .003 | -0.034 | -0.007 | 0.005 | 0.141 | -0.001 | 0.012 | -0.003 | 0.311 | -0.010 | 0.004 |
| Slope mean | -0.018 | .000 | -0.025 | -0.011 | 0.003 | 0.060 | 0.000 | 0.007 | -0.001 | 0.593 | -0.005 | 0.003 |
| Gender (male=1) | -0.007 | .078 | -0.015 | 0.001 | -0.006 | 0.005 | -0.010 | -0.002 | 0.003 | 0.149 | -0.001 | 0.008 |
| Education (high = 1) | 0.001 | .813 | -0.007 | 0.009 | 0.003 | 0.277 | -0.002 | 0.007 | -0.005 | 0.053 | -0.009 | 0.000 |
| Age linear ^*^ | 0.007 | .008 | 0.001 | 0.013 | -0.002 | 0.344 | -0.005 | 0.002 | -0.001 | 0.385 | -0.005 | 0.002 |
| Age Squared^*^ | 0.002 | .035 | 0.000 | 0.005 | 0.000 | 0.412 | -0.001 | 0.002 | 0.000 | 0.843 | -0.001 | 0.001 |
| Age Cubic^*^ | 0.001 | .060 | 0.000 | 0.002 | 0.000 | 0.757 | -0.001 | 0.001 | 0.000 | 0.748 | -0.001 | 0.001 |

*^*^ Age is grand-mean centered in the sample in these analyses to avoid multicollinearity between the age variables.*

SI Table 7.3. Results of the Latent Growth Curve Models per value using the SEM approach (N=1599)^7^ with age, age squared, and age cubic as explanatory variables and no cohort effects.^7^ In the analysis age was divided by 10 to give the variabels a comparable scale. A change of 1 unit in the coefficient here refers to a change in 10 years of age.

|  | Power | | | | Security | | | | Conformity | | | |
| --- | --- | --- | --- | --- | --- | --- | --- | --- | --- | --- | --- | --- |
|  | Coef. | P value | CI lower | CI Higher | Coef. | P value | CI lower | CI Higher | Coef. | P value | CI lower | CI Higher |
| Intercept mean | -0.905 | 0.000 | -0.971 | -0.842 | 0.575 | 0.000 | 0.531 | 0.620 | 0.092 | 0.001 | 0.042 | 0.145 |
| Gender (male=1) | 0.263 | 0.000 | 0.186 | 0.343 | -0.186 | 0.000 | -0.240 | -0.133 | -0.122 | 0.000 | -0.186 | -0.058 |
| Education (high = 1) | 0.099 | 0.019 | 0.019 | 0.183 | -0.250 | 0.000 | -0.308 | -0.193 | -0.255 | 0.000 | -0.319 | -0.191 |
| Age linear ^*^ | -0.018 | 0.524 | -0.077 | 0.037 | 0.032 | 0.079 | -0.005 | 0.069 | -0.027 | 0.199 | -0.069 | 0.016 |
| Age Squared^*^ | 0.005 | 0.605 | -0.015 | 0.024 | 0.016 | 0.020 | 0.002 | 0.029 | 0.027 | 0.001 | 0.011 | 0.042 |
| Age Cubic^*^ | 0.000 | 0.949 | -0.011 | 0.010 | 0.012 | 0.000 | 0.005 | 0.018 | 0.009 | 0.017 | 0.001 | 0.016 |
| Slope mean | -0.010 | 0.005 | -0.017 | -0.003 | 0.016 | 0.000 | 0.012 | 0.020 | 0.000 | 0.873 | -0.005 | 0.004 |
| Gender (male=1) | -0.007 | 0.090 | -0.015 | 0.001 | 0.005 | 0.042 | 0.000 | 0.009 | 0.006 | 0.030 | 0.000 | 0.012 |
| Education (high = 1) | -0.007 | 0.089 | -0.015 | 0.001 | 0.007 | 0.004 | 0.002 | 0.011 | -0.002 | 0.591 | -0.007 | 0.004 |
| Age linear ^*^ | 0.008 | 0.011 | 0.002 | 0.013 | -0.001 | 0.650 | -0.004 | 0.003 | -0.004 | 0.063 | -0.008 | 0.000 |
| Age Squared^*^ | 0.001 | 0.289 | -0.001 | 0.003 | -0.002 | 0.008 | -0.003 | -0.001 | -0.001 | 0.182 | -0.002 | 0.001 |
| Age Cubic^*^ | 0.000 | 0.715 | -0.001 | 0.001 | -0.001 | 0.004 | -0.002 | 0.000 | 0.000 | 0.236 | 0.000 | 0.001 |

*^*^ Age is grand-mean centered in the sample in these analyses to avoid multicollinearity between the age variables.*

SI Table 8.1. Results of the Latent Growth Curve Models per value using the SEM approach^7^ with age, demographics, generation, and age within each generation as explanatory variables. (N=1,599). In the table age is divided by 10 to get similar scales across variables. This means that 1 unit change in the estimate refers to a change when a person is 10 years older.

|  | Benevolence | | | | Universalism | | | | Self-direction | | | |
| --- | --- | --- | --- | --- | --- | --- | --- | --- | --- | --- | --- | --- |
|  | Coef. | P value | CI lower | CI Higher | Coef. | P value | CI lower | CI Higher | Coef. | P value | CI lower | CI Higher |
| Intercept mean | 0.485 | 0.000 | 0.434 | 0.538 | 0.485 | 0.000 | 0.434 | 0.538 | 0.434 | 0.000 | 0.377 | 0.493 |
| Gender (male=1) | -0.201 | 0.000 | -0.244 | -0.163 | -0.201 | 0.000 | -0.244 | -0.163 | -0.061 | 0.012 | -0.110 | -0.016 |
| Education (high = 1) | 0.092 | 0.000 | 0.050 | 0.134 | 0.092 | 0.000 | 0.050 | 0.134 | 0.173 | 0.000 | 0.118 | 0.225 |
| Age GroupMC* | 0.072 | 0.209 | -0.043 | 0.185 | 0.072 | 0.209 | -0.043 | 0.185 | 0.067 | 0.269 | -0.065 | 0.184 |
| Baby-boomers | -0.108 | 0.000 | -0.160 | -0.057 | -0.108 | 0.000 | -0.160 | -0.057 | -0.014 | 0.626 | -0.069 | 0.043 |
| Generation-X | -0.323 | 0.000 | -0.390 | -0.260 | -0.323 | 0.000 | -0.390 | -0.260 | -0.107 | 0.004 | -0.183 | -0.036 |
| Millennials | -0.499 | 0.000 | -0.593 | -0.404 | -0.499 | 0.000 | -0.593 | -0.404 | -0.392 | 0.000 | -0.497 | -0.293 |
| Baby-boomers_Age | 0.067 | 0.289 | -0.057 | 0.186 | 0.067 | 0.289 | -0.057 | 0.186 | -0.048 | 0.492 | -0.179 | 0.100 |
| Generation-X_Age | 0.002 | 0.985 | -0.152 | 0.142 | 0.002 | 0.985 | -0.152 | 0.142 | -0.087 | 0.314 | -0.253 | 0.093 |
| Millennials_Age | -0.150 | 0.236 | -0.390 | 0.104 | -0.150 | 0.236 | -0.390 | 0.104 | 0.099 | 0.434 | -0.145 | 0.351 |
| Slope mean | -0.001 | 0.543 | -0.006 | 0.003 | -0.001 | 0.543 | -0.006 | 0.003 | -0.005 | 0.042 | -0.010 | 0.000 |
| Gender (male=1) | 0.002 | 0.348 | -0.002 | 0.005 | 0.002 | 0.348 | -0.002 | 0.005 | 0.000 | 0.940 | -0.004 | 0.004 |
| Education (high = 1) | 0.002 | 0.161 | -0.001 | 0.006 | 0.002 | 0.161 | -0.001 | 0.006 | 0.001 | 0.728 | -0.003 | 0.005 |
| Age GroupMC | -0.011 | 0.018 | -0.021 | -0.002 | -0.011 | 0.018 | -0.021 | -0.002 | -0.007 | 0.211 | -0.017 | 0.004 |
| Baby-boomers | 0.006 | 0.011 | 0.001 | 0.010 | 0.006 | 0.011 | 0.001 | 0.010 | 0.009 | 0.000 | 0.004 | 0.014 |
| Generation-X | 0.011 | 0.000 | 0.006 | 0.016 | 0.011 | 0.000 | 0.006 | 0.016 | 0.014 | 0.000 | 0.007 | 0.020 |
| Millennials | 0.018 | 0.000 | 0.009 | 0.027 | 0.018 | 0.000 | 0.009 | 0.027 | 0.022 | 0.000 | 0.012 | 0.031 |
| Baby-boomers_Age | 0.009 | 0.101 | -0.002 | 0.019 | 0.009 | 0.101 | -0.002 | 0.019 | 0.001 | 0.842 | -0.011 | 0.013 |
| Generation-X_Age | 0.004 | 0.585 | -0.009 | 0.015 | 0.004 | 0.585 | -0.009 | 0.015 | 0.004 | 0.642 | -0.012 | 0.018 |
| Millennials_Age | 0.022 | 0.092 | -0.005 | 0.048 | 0.022 | 0.092 | -0.005 | 0.048 | -0.003 | 0.807 | -0.033 | 0.024 |

*^*^Age is group-mean centered to avoid multicollinearity with the cohort variable. ^**^Silent-generation is the reference category.*

SI Table 8.2. Results of the Latent Growth Curve Models per value using the SEM approach^7^ with age, demographics, generation, and age within each generation as explanatory variables. (N=1,599). In the table age is divided by 10 to get similar scales across variables. This means that 1 unit change in the estimate refers to a change when a person is 10 years older.

|  | Stimulation | | | | Hedonism | | | | Achievement | | | |
| --- | --- | --- | --- | --- | --- | --- | --- | --- | --- | --- | --- | --- |
|  | Coef. | P value | CI lower | CI Higher | Coef. | P value | CI lower | CI Higher | Coef. | P value | CI lower | CI Higher |
| Intercept mean | -1.392 | 0.000 | -1.509 | -1.278 | 0.051 | 0.086 | -0.005 | 0.112 | -0.326 | 0.000 | -0.393 | -0.264 |
| Gender (male=1) | 0.391 | 0.000 | 0.306 | 0.479 | 0.013 | 0.575 | -0.034 | 0.058 | 0.090 | 0.000 | 0.041 | 0.141 |
| Education (high = 1) | -0.139 | 0.006 | -0.241 | -0.040 | -0.029 | 0.259 | -0.081 | 0.021 | 0.258 | 0.000 | 0.205 | 0.305 |
| Age GroupMC | -0.490 | 0.003 | -0.810 | -0.152 | -0.159 | 0.014 | -0.281 | -0.016 | 0.022 | 0.749 | -0.106 | 0.157 |
| Baby-boomers | 0.279 | 0.000 | 0.165 | 0.393 | 0.192 | 0.000 | 0.130 | 0.250 | -0.161 | 0.000 | -0.218 | -0.097 |
| Generation-X | 0.517 | 0.000 | 0.376 | 0.672 | 0.435 | 0.000 | 0.353 | 0.503 | -0.306 | 0.000 | -0.383 | -0.225 |
| Millennials | 0.974 | 0.000 | 0.749 | 1.186 | 0.555 | 0.000 | 0.448 | 0.665 | -0.269 | 0.000 | -0.378 | -0.160 |
| Baby-boomers_Age | 0.323 | 0.059 | -0.022 | 0.648 | 0.016 | 0.821 | -0.133 | 0.150 | 0.038 | 0.619 | -0.115 | 0.179 |
| Generation-X_Age | 0.332 | 0.106 | -0.086 | 0.714 | -0.061 | 0.525 | -0.259 | 0.127 | -0.188 | 0.046 | -0.363 | 0.000 |
| Millennials_Age | -0.357 | 0.290 | -1.007 | 0.351 | 0.327 | 0.033 | 0.020 | 0.632 | 0.141 | 0.384 | -0.175 | 0.482 |
| Slope mean | 0.008 | 0.155 | -0.003 | 0.019 | 0.001 | 0.654 | -0.004 | 0.007 | -0.003 | 0.251 | -0.009 | 0.002 |
| Gender (male=1) | -0.007 | 0.092 | -0.014 | 0.001 | -0.006 | 0.007 | -0.010 | -0.002 | 0.003 | 0.150 | -0.001 | 0.008 |
| Education (high = 1) | 0.001 | 0.781 | -0.007 | 0.009 | 0.002 | 0.327 | -0.003 | 0.007 | -0.004 | 0.066 | -0.009 | 0.000 |
| Baby-boomers | 0.042 | 0.001 | 0.015 | 0.067 | 0.007 | 0.276 | -0.006 | 0.019 | 0.005 | 0.425 | -0.007 | 0.016 |
| Generation-X | -0.021 | 0.000 | -0.032 | -0.010 | 0.003 | 0.304 | -0.003 | 0.008 | 0.002 | 0.575 | -0.004 | 0.007 |
| Millennials | -0.033 | 0.000 | -0.046 | -0.020 | 0.004 | 0.291 | -0.003 | 0.011 | 0.005 | 0.203 | -0.003 | 0.013 |
| Age GroupMC | -0.054 | 0.000 | -0.075 | -0.033 | 0.009 | 0.154 | -0.004 | 0.021 | 0.005 | 0.408 | -0.007 | 0.018 |
| Baby-boomers_Age | -0.036 | 0.009 | -0.063 | -0.006 | -0.008 | 0.228 | -0.021 | 0.007 | -0.005 | 0.416 | -0.017 | 0.008 |
| Generation-X_Age | -0.028 | 0.105 | -0.063 | 0.007 | -0.007 | 0.399 | -0.023 | 0.011 | 0.010 | 0.238 | -0.007 | 0.028 |
| Millennials_Age | -0.049 | 0.143 | -0.116 | 0.012 | -0.015 | 0.413 | -0.050 | 0.023 | -0.016 | 0.417 | -0.057 | 0.024 |

*^*^Age is group-mean centered to avoid multicollinearity with the cohort variable. ^**^Silent-generation is the reference category.*

SI Table 8.3. Results of the Latent Growth Curve Models per value using the SEM approach^7^ with age, demographics, generation, and age within each generation as explanatory variables. (N=1,599). In the table age is divided by 10 to get similar scales across variables. This means that 1 unit change in the estimate refers to a change when a person is 10 years older.

|  | Power | | | | Security | | | | Conformity | | | |
| --- | --- | --- | --- | --- | --- | --- | --- | --- | --- | --- | --- | --- |
|  | Coef. | P value | CI lower | CI Higher | Coef. | P value | CI lower | CI Higher | Coef. | P value | CI lower | CI Higher |
| Intercept mean | -0.925 | 0.000 | -1.027 | -0.820 | 0.743 | 0.000 | 0.680 | 0.803 | 0.162 | 0.000 | 0.087 | 0.241 |
| Gender (male=1) | 0.264 | 0.000 | 0.181 | 0.344 | -0.186 | 0.000 | -0.237 | -0.136 | -0.123 | 0.000 | -0.191 | -0.060 |
| Education (high = 1) | 0.094 | 0.028 | 0.010 | 0.179 | -0.247 | 0.000 | -0.305 | -0.190 | -0.251 | 0.000 | -0.320 | -0.184 |
| Age GroupMC* | 0.090 | 0.364 | -0.109 | 0.290 | 0.237 | 0.001 | 0.087 | 0.373 | 0.214 | 0.010 | 0.030 | 0.367 |
| Baby-boomers | 0.025 | 0.633 | -0.078 | 0.126 | -0.154 | 0.000 | -0.220 | -0.091 | -0.073 | 0.067 | -0.151 | 0.004 |
| Generation-X | 0.014 | 0.827 | -0.117 | 0.136 | -0.199 | 0.000 | -0.281 | -0.117 | 0.023 | 0.631 | -0.069 | 0.120 |
| Millennials | 0.201 | 0.027 | 0.017 | 0.376 | -0.433 | 0.000 | -0.563 | -0.316 | 0.001 | 0.987 | -0.147 | 0.149 |
| Baby-boomers_Age | -0.161 | 0.167 | -0.389 | 0.070 | -0.162 | 0.038 | -0.307 | -0.001 | -0.153 | 0.097 | -0.324 | 0.047 |
| Generation-X_Age | 0.025 | 0.876 | -0.288 | 0.338 | -0.019 | 0.851 | -0.217 | 0.180 | -0.071 | 0.539 | -0.300 | 0.168 |
| Millennials_Age | 0.145 | 0.563 | -0.381 | 0.616 | -0.076 | 0.631 | -0.390 | 0.223 | -0.288 | 0.117 | -0.649 | 0.057 |
| Slope mean | 0.007 | 0.114 | -0.001 | 0.017 | 0.003 | 0.301 | -0.003 | 0.009 | -0.005 | 0.139 | -0.011 | 0.002 |
| Gender (male=1) | -0.007 | 0.093 | -0.015 | 0.001 | 0.005 | 0.037 | 0.000 | 0.009 | 0.006 | 0.044 | 0.000 | 0.011 |
| Education (high = 1) | -0.007 | 0.113 | -0.015 | 0.002 | 0.007 | 0.006 | 0.002 | 0.011 | -0.002 | 0.526 | -0.008 | 0.003 |
| Baby-boomers | 0.011 | 0.214 | -0.006 | 0.030 | -0.018 | 0.004 | -0.030 | -0.006 | -0.016 | 0.027 | -0.029 | -0.002 |
| Generation-X | -0.015 | 0.004 | -0.024 | -0.004 | 0.012 | 0.000 | 0.006 | 0.018 | 0.002 | 0.561 | -0.005 | 0.009 |
| Millennials | -0.024 | 0.000 | -0.038 | -0.012 | 0.011 | 0.002 | 0.004 | 0.019 | 0.007 | 0.104 | -0.002 | 0.016 |
| Age GroupMC* | -0.043 | 0.000 | -0.062 | -0.022 | 0.027 | 0.000 | 0.015 | 0.041 | -0.001 | 0.847 | -0.016 | 0.013 |
| Baby-boomers_Age | -0.003 | 0.782 | -0.024 | 0.018 | 0.015 | 0.029 | 0.002 | 0.029 | 0.015 | 0.047 | 0.000 | 0.031 |
| Generation-X_Age | -0.022 | 0.174 | -0.055 | 0.008 | 0.012 | 0.177 | -0.005 | 0.030 | 0.011 | 0.328 | -0.013 | 0.035 |
| Millennials_Age | -0.042 | 0.133 | -0.097 | 0.012 | 0.023 | 0.226 | -0.014 | 0.061 | 0.077 | 0.000 | 0.033 | 0.120 |

*^*^Age is group-mean centered to avoid multicollinearity with the cohort variable. ^**^Silent-generation is the reference category.*

SI Table 9.1. Results of the Latent Growth Curve Models per value using the SEM approach^7^ with age, age squared, and age cubic as well as demographics and generation as explanatory variables. Based on the sample (N=2,033) including missing observations at one or more waves between 2008 and 2020.

|  | Benevolence | | | | Universalism | | | | Self-direction | | | |
| --- | --- | --- | --- | --- | --- | --- | --- | --- | --- | --- | --- | --- |
|  | Coef | P value | CI lower | CI Higher | Coef. | P value | CI lower | CI Higher | Coef. | P value | CI lower | CI Higher |
| Intercept mean | 0.742 | .000 | 0.687 | 0.804 | 0.480 | .000 | 0.425 | 0.530 | 0.425 | .000 | 0.366 | 0.481 |
| Gender (male=1) | -0.154 | .000 | -0.196 | -0.112 | -0.179 | .000 | -0.216 | -0.145 | -0.046 | .036 | -0.088 | -0.001 |
| Education (high = 1) | 0.040 | .070 | -0.003 | 0.083 | 0.068 | .000 | 0.033 | 0.105 | 0.163 | .000 | 0.121 | 0.212 |
| Age linear ^*^ | -0.005 | .906 | -0.073 | 0.091 | 0.092 | .007 | 0.011 | 0.150 | 0.027 | .515 | -0.067 | 0.093 |
| Age Squared^*^ | 0.010 | .817 | -0.078 | 0.094 | -0.045 | .236 | -0.115 | 0.033 | 0.000 | 1.000 | -0.087 | 0.097 |
| Age Cubic^*^ | 0.039 | .567 | -0.141 | 0.133 | -0.004 | .942 | -0.079 | 0.135 | -0.022 | .753 | -0.122 | 0.141 |
| Baby-boomers^1^ | 0.021 | .489 | -0.041 | 0.082 | -0.095 | .000 | -0.145 | -0.042 | -0.002 | .962 | -0.062 | 0.065 |
| Generation-X | -0.051 | .149 | -0.128 | 0.017 | -0.316 | .000 | -0.372 | -0.257 | -0.111 | .001 | -0.177 | -0.047 |
| Millennials | -0.112 | .008 | -0.197 | -0.031 | -0.440 | .000 | -0.514 | -0.361 | -0.345 | .000 | -0.438 | -0.257 |
| Slope mean | -0.004 | .177 | -0.008 | 0.001 | 0.000 | .906 | -0.004 | 0.004 | -0.003 | .182 | -0.008 | 0.002 |
| Gender (male=1) | 0.003 | .118 | -0.001 | 0.006 | 0.000 | .849 | -0.003 | 0.003 | -0.001 | .459 | -0.005 | 0.002 |
| Education (high = 1) | 0.001 | .589 | -0.003 | 0.005 | 0.004 | .015 | 0.001 | 0.007 | 0.001 | .743 | -0.003 | 0.004 |
| Age linear ^*^ | 0.001 | .866 | -0.006 | 0.007 | 0.001 | .734 | -0.004 | 0.007 | -0.001 | .687 | -0.007 | 0.006 |
| Age Squared^*^ | -0.007 | .041 | -0.014 | 0.000 | -0.003 | .417 | -0.009 | 0.004 | -0.002 | .623 | -0.009 | 0.006 |
| Age Cubic^*^ | -0.005 | .220 | -0.014 | 0.004 | -0.005 | .237 | -0.014 | 0.003 | -0.002 | .620 | -0.014 | 0.006 |
| Baby-boomers | 0.003 | .189 | -0.002 | 0.009 | 0.006 | .005 | 0.002 | 0.010 | 0.008 | .003 | 0.003 | 0.013 |
| Generation-X | 0.005 | .070 | 0.000 | 0.011 | 0.010 | .000 | 0.005 | 0.015 | 0.014 | .000 | 0.008 | 0.019 |
| Millennials | 0.016 | .000 | 0.008 | 0.024 | 0.019 | .000 | 0.011 | 0.026 | 0.022 | .000 | 0.014 | 0.031 |

*^*^Age is group-mean centered to avoid multicollinearity with the cohort variable. ^**^Silent-generation is the reference category.*

SI Table 9.2. LGCM with age, age squared, and age cubic as well as demographics and generation as explanatory variables. Table is based on the sample (N=2,033) including missing observations at one or more waves between 2008 and 2020.

|  | Stimulation | | | | Hedonism | | | | Achievement | | | |
| --- | --- | --- | --- | --- | --- | --- | --- | --- | --- | --- | --- | --- |
|  | Coef. | P value | CI lower | CI Higher | Coef. | P value | CI lower | CI Higher | Coef. | P value | CI lower | CI Higher |
| Intercept mean | -1.331 | .000 | -1.449 | 0.481 | 0.078 | .008 | 0.019 | 0.136 | -0.330 | .000 | -0.393 | -0.272 |
| Gender (male=1) | 0.389 | .000 | 0.317 | 0.468 | 0.007 | .756 | -0.039 | 0.049 | 0.080 | .001 | 0.034 | 0.125 |
| Education (high = 1) | -0.141 | .001 | -0.229 | -0.061 | -0.047 | .043 | -0.096 | -0.003 | 0.262 | .000 | 0.212 | 0.311 |
| Age linear ^*^ | -0.209 | .004 | -0.376 | -0.079 | -0.140 | .000 | -0.211 | -0.052 | 0.022 | .571 | -0.075 | 0.088 |
| Age Squared^*^ | -0.182 | .034 | -0.342 | -0.004 | -0.011 | .801 | -0.098 | 0.078 | 0.033 | .506 | -0.062 | 0.129 |
| Age Cubic^*^ | -0.021 | .863 | -0.206 | 0.289 | 0.019 | .776 | -0.142 | 0.114 | 0.020 | .752 | -0.073 | 0.184 |
| Baby-boomers^1^ | 0.299 | .000 | 0.174 | 0.411 | 0.174 | .000 | 0.112 | 0.235 | -0.150 | .000 | -0.207 | -0.089 |
| Generation-X | 0.518 | .000 | 0.389 | 0.645 | 0.396 | .000 | 0.322 | 0.463 | -0.248 | .000 | -0.317 | -0.179 |
| Millennials | 1.033 | .000 | 0.870 | 1.225 | 0.481 | .000 | 0.390 | 0.575 | -0.200 | .000 | -0.282 | -0.112 |
| Slope mean | 0.006 | .260 | -0.005 | 0.015 | -0.001 | .630 | -0.007 | 0.004 | -0.003 | .289 | -0.008 | 0.003 |
| Gender (male=1) | -0.006 | .090 | -0.013 | 0.001 | -0.005 | .026 | -0.009 | 0.000 | 0.003 | .118 | -0.001 | 0.007 |
| Education (high = 1) | 0.002 | .510 | -0.005 | 0.009 | 0.002 | .305 | -0.002 | 0.007 | -0.006 | .007 | -0.010 | -0.002 |
| Age linear ^*^ | 0.006 | .377 | -0.009 | 0.017 | -0.004 | .190 | -0.011 | 0.002 | -0.001 | .857 | -0.007 | 0.007 |
| Age Squared^*^ | 0.001 | .885 | -0.014 | 0.015 | 0.006 | .165 | -0.002 | 0.014 | 0.004 | .368 | -0.005 | 0.012 |
| Age Cubic^*^ | 0.009 | .417 | -0.008 | 0.034 | 0.003 | .590 | -0.007 | 0.013 | 0.002 | .751 | -0.012 | 0.011 |
| Baby-boomers | -0.019 | .000 | -0.029 | -0.008 | 0.004 | .173 | -0.002 | 0.009 | 0.001 | .712 | -0.004 | 0.006 |
| Generation-X | -0.030 | .000 | -0.042 | -0.018 | 0.004 | .175 | -0.002 | 0.011 | 0.002 | .490 | -0.004 | 0.009 |
| Millennials | -0.060 | .000 | -0.077 | -0.043 | 0.011 | .028 | 0.001 | 0.020 | -0.001 | .846 | -0.011 | 0.009 |

*^*^Age is group-mean centered to avoid multicollinearity with the cohort variable. ^**^Silent-generation is the reference category.*

SI Table 9.3. LGCM with age, age squared, and age cubic as well as demographics and generation as explanatory variables. Table is based on the sample (N=2,033) including missing observations at one or more waves between 2008 and 2020.

|  | Power | | | | Security | | | | Conformity | | | |
| --- | --- | --- | --- | --- | --- | --- | --- | --- | --- | --- | --- | --- |
|  | Coef. | P value | CI lower | CI Higher | Coef. | P value | CI lower | CI Higher | Coef. | P value | CI lower | CI Higher |
| Intercept mean | -0.907 | .000 | -1.013 | -0.804 | 0.715 | .000 | 0.660 | 0.779 | 0.127 | .001 | 0.053 | 0.203 |
| Gender (male=1) | 0.229 | .000 | 0.153 | 0.306 | -0.203 | .000 | -0.248 | -0.157 | -0.123 | .000 | -0.180 | -0.067 |
| Education (high = 1) | 0.131 | .000 | 0.058 | 0.205 | -0.229 | .000 | -0.278 | -0.177 | -0.248 | .000 | -0.311 | -0.191 |
| Age linear ^*^ | 0.015 | .800 | -0.106 | 0.136 | 0.130 | .001 | 0.065 | 0.221 | 0.068 | .161 | -0.023 | 0.167 |
| Age Squared^*^ | -0.066 | .413 | -0.223 | 0.093 | 0.119 | .011 | 0.026 | 0.209 | 0.143 | .011 | 0.029 | 0.257 |
| Age Cubic^*^ | -0.009 | .921 | -0.197 | 0.163 | -0.028 | .653 | -0.187 | 0.063 | 0.006 | .934 | -0.153 | 0.132 |
| Baby-boomers^1^ | 0.040 | .451 | -0.059 | 0.148 | -0.186 | .000 | -0.256 | -0.120 | -0.102 | .011 | -0.185 | -0.025 |
| Generation-X | 0.048 | .407 | -0.056 | 0.169 | -0.247 | .000 | -0.322 | -0.178 | 0.011 | .806 | -0.082 | 0.096 |
| Millennials | 0.121 | .120 | -0.030 | 0.269 | -0.454 | .000 | -0.554 | -0.351 | -0.081 | .189 | -0.203 | 0.040 |
| Slope mean | 0.003 | .516 | -0.006 | 0.012 | 0.004 | .161 | -0.002 | 0.009 | -0.001 | .696 | -0.008 | 0.005 |
| Gender (male=1) | -0.005 | .227 | -0.012 | 0.003 | 0.006 | .007 | 0.002 | 0.010 | 0.004 | .093 | -0.001 | 0.010 |
| Education (high = 1) | -0.006 | .121 | -0.013 | 0.002 | 0.004 | .060 | 0.000 | 0.008 | -0.002 | .433 | -0.007 | 0.003 |
| Age linear ^*^ | 0.000 | .984 | -0.013 | 0.011 | -0.005 | .187 | -0.011 | 0.002 | 0.004 | .352 | -0.004 | 0.013 |
| Age Squared^*^ | 0.010 | .212 | -0.006 | 0.025 | -0.005 | .294 | -0.013 | 0.004 | -0.005 | .335 | -0.015 | 0.005 |
| Age Cubic^*^ | 0.006 | .482 | -0.010 | 0.026 | 0.002 | .637 | -0.009 | 0.012 | -0.009 | .135 | -0.022 | 0.001 |
| Baby-boomers | -0.014 | .005 | -0.023 | -0.004 | 0.012 | .000 | 0.006 | 0.018 | -0.001 | .862 | -0.007 | 0.006 |
| Generation-X | -0.025 | .000 | -0.035 | -0.013 | 0.013 | .000 | 0.007 | 0.019 | 0.006 | .117 | -0.002 | 0.014 |
| Millennials | -0.037 | .000 | -0.054 | -0.021 | 0.024 | .000 | 0.013 | 0.034 | 0.006 | .343 | -0.006 | 0.019 |

*^*^Age is group-mean centered to avoid multicollinearity with the cohort variable. ^**^Silent-generation is the reference category.*

SI 12: SI Table 10-13. Comparing value differences between cohorts (with similar age) and within cohort (the same people in T0, 2008 and T6, 2020).

SI Table 10: T-test results for the sample of people without missing values (1599 respondents) We used a Between Subject T-test (comparing different people with similar age). Cohorts based on age in 2008 16:28='1'; 29:40='2'; 41:52='3'; 53:64='4'; 65:90='5'.

|  |  | **Ben** |  | **Uni** |  | **Sdi** |  | **Sti** |  | **Hed** |  | **Ach** |  | **Pow** |  | **Sec** |  | **Con** |  |
| --- | --- | --- | --- | --- | --- | --- | --- | --- | --- | --- | --- | --- | --- | --- | --- | --- | --- | --- | --- |
| **COMPARING BETWEEN COHORTS**  A* | B | Mean Diff. (B-A)* | p | Mean Diff. (B-A)* | p | Mean Diff. (B-A)* | p | Mean Diff. (B-A)* | p | Mean Diff. (B-A)* | p | Mean Diff. (B-A)* | p | Mean Diff. (B-A)* | p | Mean Diff. (B-A)* | p | Mean Diff. (B-A)* | P |
| 2008 people 29-40 **(2)** | 2020 people 29-40 **(1)** | -0.005 | 0.938 | -0.132 | 0.040 | -0.010 | 0.878 | 0.173 | 0.198 | -0.165 | 0.033 | -0.057 | 0.450 | 0.154 | 0.241 | -0.136 | 0.075 | 0.178 | 0.062 |
| 2008 people 41-52 **(3)** | 2020 people 41-52 **(2)** | -0.002 | 0.964 | -0.058 | 0.154 | -0.065 | 0.180 | 0.169 | 0.050 | -0.292 | 0.000 | -0.292 | 0.000 | 0.382 | 0.000 | -0.177 | 0.000 | -0.060 | 0.344 |
| 2008 people 53-64 **(4)** | 2020 people 53-64 **(3)** | -0.016 | 0.644 | -0.060 | 0.094 | -0.060 | 0.094 | 0.135 | 0.040 | -0.162 | 0.000 | -0.162 | 0.000 | 0.218 | 0.002 | -0.252 | 0.000 | 0.037 | 0.443 |
| 2008 people 65-83 **(5)** | 2020 people 65-83 **(4)** | 0.005 | 0.902 | 0.018 | 0.619 | 0.010 | 0.814 | 0.165 | 0.062 | 0.116 | 0.007 | -0.124 | 0.011 | 0.136 | 0.067 | 0.045 | 0.320 | -0.099 | 0.086 |

* The year is the year of the measurement of the values. **Indicated age is age of the cohort in that specific year.** Number between brackets (1-5) indicates cohort.

SI Table 11. T-test results for the sample of people without missing values (1599 respondents). We used a Within subject T-test (comparing the same people with different ages). Cohorts based on age in 2008 16:28='1'; 29:40='2'; 41:52='3'; 53:64='4'; 65:90='5'.

|  |  | **Ben** |  | **Uni** |  | **Sdi** |  | **Sti** |  | **Hed** |  | **Ach** |  | **Pow** |  | **Sec** |  | **Con** |  |
| --- | --- | --- | --- | --- | --- | --- | --- | --- | --- | --- | --- | --- | --- | --- | --- | --- | --- | --- | --- |
| **COMPARING**  **WITHIN COHORTS**  A* | B | Mean Diff. (B-A)* | P | Mean Diff. (B-A)* | p | Mean Diff. (B-A)* | p | Mean Diff. (B-A)* | p | Mean Diff. (B-A)* | p | Mean Diff. (B-A)* | p | Mean Diff. (B-A)* | p | Mean Diff. (B-A)* | p | Mean Diff. (B-A)* | p |
| 2008 people 16-28 (1) | 2020 people 28-34 (1) | -0.126 | 0.075 | -0.230 | 0.000 | -0.186 | 0.004 | 0.555 | 0.000 | -0.101 | 0.213 | -0.030 | 0.720 | 0.376 | 0.004 | -0.404 | 0.000 | 0.145 | 0.107 |
| 2008 people 29-40 (2) | 2020 people 29-40 (2) | -0.035 | 0.369 | -0.183 | 0.000 | -0.119 | 0.002 | 0.336 | 0.000 | -0.050 | 0.239 | 0.048 | 0.329 | 0.264 | 0.001 | -0.244 | 0.000 | -0.016 | 0.760 |
| 2008 people 41-52 (3) | 2020 people 41-52 (3) | 0.386 | 0.022 | -0.104 | 0.000 | -0.081 | 0.000 | 0.221 | 0.000 | 0.221 | 0.022 | 0.000 | 0.998 | 0.245 | 0.000 | -0.265 | 0.000 | 0.029 | 0.461 |
| 2008 people 53-64 (5) | 2020 people 53-64 (4) | 0.875 | 0.004 | -0.070 | 0.000 | -0.030 | 0.173 | 0.139 | 0.001 | 0.007 | 0.782 | 0.039 | 0.126 | 0.132 | 0.006 | -0.222 | 0.000 | 0.002 | 0.936 |
| 2008 people 65+ (5) | 2020 people 77+ (5) | 0.059 | 0.011 | 0.715 | 0.286 | 0.085 | 0.019 | -0.148 | 0.081 | 0.014 | 0.742 | 0.005 | 0.906 | -0.052 | 0.444 | -0.046 | 0.278 | 0.073 | 0.174 |

* The year is the year of the measurement of the values. Indicated age is age of the cohort in that specific year**.** Number between brackets (1-5) indicates cohort.

SI Table 12. T-test results for the sample of people including those with missing values (2033 respondents) We used a Between Subject T-test (comparing different people with similar age). Cohorts based on age in 2008 16:28='1'; 29:40='2'; 41:52='3'; 53:64='4'; 65:90='5'. Number of observations per cohort: 1:106, 2:250. 3:456, 4:567, 5:220.

|  |  | **Ben** |  | **Uni** |  | **Sdi** |  | **Sti** |  | **Hed** |  | **Ach** |  | **Pow** |  | **Sec** |  | **Con** |  |
| --- | --- | --- | --- | --- | --- | --- | --- | --- | --- | --- | --- | --- | --- | --- | --- | --- | --- | --- | --- |
| **COMPARING BETWEEN COHORTS**  A* | `  B | Mean Diff. (B-A)* | P | Mean Diff. (B-A)* | p | Mean Diff. (B-A)* | P | Mean Diff. (B-A)* | p | Mean Diff. (B-A)* | p | Mean Diff. (B-A)* | p | Mean Diff. (B-A)* | p | Mean Diff. (B-A)* | p | Mean Diff. (B-A)* | p |
| 2020 people 29-40 (1) | 2008 people 29-40 (2) | 0.076 | .137 | 0.177 | .000 | 0.050 | .332 | -0.195 | .063 | 0.178 | .002 | -0.001 | .982 | -0.299 | .004 | 0.104 | .071 | -0.088 | .230 |
| 2020 people 41-52 (2) | 2008 people 41-52 (3) | -0.032 | .378 | 0.034 | .317 | 0.029 | .463 | -0.126 | .076 | 0.256 | .000 | -0.125 | .004 | -0.307 | .000 | 0.156 | .000 | 0.115 | .023 |
| 2020 people 53-64 (3) | 2008 people 53-64 (4) | 0.007 | .816 | -0.029 | .289 | 0.051 | .118 | -0.117 | .048 | 0.185 | .000 | -0.056 | .096 | -0.197 | .002 | 0.211 | .000 | -0.056 | .203 |
| 2020 people 65-83 (4) | 2008 people 65-83 (5) | 0.003 | .940 | 0.017 | .619 | 0.019 | .632 | 0.192 | .021 | 0.093 | .026 | -0.103 | .024 | -0.138 | .053 | 0.022 | .613 | -0.104 | .061 |

* The year is the year of the measurement of the values. **Indicated age is age of the cohort in that specific year.** Number between brackets (1-5) indicates cohort.

SI Table 13. T-test results for the sample of people including those with missing values (2033 respondents). We used a Within subject T-test (comparing the same people with different ages). Cohorts based on age in 2008 16:28='1'; 29:40='2'; 41:52='3'; 53:64='4'; 65:90='5'. Number of observations per cohort: 1:187, 2:378. 3:578, 4:651, 5:239

|  |  | **Ben** |  | **Uni** |  | **Sdi** |  | **Sti** |  | **Hed** |  | **Ach** |  | **Pow** |  | **Sec** |  | **Con** |  |
| --- | --- | --- | --- | --- | --- | --- | --- | --- | --- | --- | --- | --- | --- | --- | --- | --- | --- | --- | --- |
| **COMPARING WITHIN COHORTS**  A* | B | Mean Diff. (B-A)* | p | Mean Diff. (B-A)* | p | Mean Diff. (B-A)* | p | Mean Diff. (B-A)* | p | Mean Diff. (B-A)* | p | Mean Diff. (B-A)* | p | Mean Diff. (B-A)* | p | Mean Diff. (B-A)* | p | Mean Diff. (B-A)* | p |
| 2008 people 16-28 (1) | 2020 people 28-34 (1) | 0.149 | .002 | 0.222 | .000 | 0.199 | .000 | -0.635 | .000 | 0.135 | .023 | -0.045 | .490 | -0.368 | .000 | 0.336 | .000 | 0.007 | .926 |
| 2008 people 29-40 (2) | 2020 people 29-40 (2) | 0.031 | .322 | 0.171 | .000 | 0.133 | .000 | -0.319 | .000 | 0.050 | .168 | -0.055 | .162 | -0.290 | .000 | 0.219 | .000 | 0.059 | .157 |
| 2008 people 41-52 (3) | 2020 people 41-52 (3) | -0.043 | .055 | 0.093 | .000 | 0.049 | .047 | -0.190 | .000 | 0.082 | .002 | 0.005 | .849 | -0.195 | .000 | 0.239 | .000 | -0.040 | .256 |
| 2008 people 53-64 (5) | 2020 people 53-64 (4) | 0.004 | .844 | 0.069 | .000 | 0.041 | .046 | -0.129 | .001 | -0.011 | .627 | -0.030 | .216 | -0.147 | .001 | 0.208 | .000 | -0.006 | .831 |
| 2008 people 65+ (5) | 2020 people 77+ (5) | -0.077 | .045 | -0.008 | .783 | -0.082 | .016 | 0.148 | .062 | -0.007 | .864 | -0.004 | .926 | 0.056 | .392 | 0.032 | .426 | -0.059 | .253 |

* The year is the year of the measurement of the values. Indicated age is age of the cohort in that specific year. Number between brackets (1-5) indicates cohort.

SI 13: SI Table 14. Sample composition in the main sample of our study, the full sample of LISS in 2008 and the sample covering 2008-2020 with missing observations.

|  | Main sample (all waves present) | | Full LISS sample in 2008 | | Sample of people present in 2008 and 2020 with missing on in-between waves of LISS | |
| --- | --- | --- | --- | --- | --- | --- |
|  | 2008-2020 |  |  |  | 2008-2020 |  |
|  | N=1599 | % | N=6700 | % | N=2033 | % |
| Male | 787 | 49,2 | 3065 | 45,8 | 979 | 48,2 |
| Female | 812 | 50,8 | 3635 | 54,2 | 1054 | 51,8 |
| Low education | 1025 | 64,1 | 5469 | 81,6 | 1265 | 62,2 |
| High education | 574 | 35,9 | 1231 | 18,4 | 768 | 37,8 |
| Silent generation | 285 | 17,8 | 1128 | 16,8 | 313 | 15,4 |
| Babyboom generation | 865 | 54,1 | 2681 | 40 | 1029 | 50,6 |
| Generation X | 343 | 21,5 | 1856 | 27,7 | 504 | 24,8 |
| Millennial generation | 106 | 6,6 | 1035 | 15,5 | 187 | 9,2 |
| Average Year of Birth | 1957,45 |  | 1961,63 |  | 1959,34 |  |

SI 14: Reference list supplementary materials.

1 Van Herk, H., Schoonees, P. C., Groenen, P. J. & van Rosmalen, J. Competing for the same value segments? Insight into the volatile Dutch political landscape. *PloS one* **13**, e0190598 (2018).

2 Schwartz, S. H. *et al.* Refining the theory of basic individual values. *Journal of personality and social psychology* **103**, 663 (2012).

3 De Leeuw, J. & Mair, P. Multidimensional scaling using majorization: SMACOF in R. *Journal of statistical software* **31**, 1-30 (2009).

4 Mair, P., Groenen, P. J. & de Leeuw, J. More on multidimensional scaling and unfolding in R: smacof version 2. *Journal of Statistical Software* **102**, 1-47 (2022).

5 Borg, I., Groenen, P. J. & Mair, P. *Applied multidimensional scaling and unfolding*.13(Springer, 2018).

6 Borg, I. & Leutner, D. Measuring the similarity of MDS configurations. *Multivariate Behavioral Research* **20**, 325-334 (1985).

7 Rosseel, Y. Lavaan: An R package for structural equation modeling and more. Version 0.5–12 (BETA). *Journal of statistical software* **48**, 1-36 (2012).
